# Contrasting global genetic patterns in two biologically similar, widespread and invasive *Ciona* species (Tunicata, Ascidiacea)

**DOI:** 10.1038/srep24875

**Published:** 2016-05-03

**Authors:** Sarah Bouchemousse, John D. D. Bishop, Frédérique Viard

**Affiliations:** 1 Sorbonne Universités, UPMC Univ Paris 6, CNRS, UMR 7144, Equipe Div&Co, Station Biologique de Roscoff, Place Georges Teissier, 29680 Roscoff, France; 2 Marine Biological Association, Citadel Hill, Plymouth PL1 2PB, UK

## Abstract

Human-mediated dispersal interplays with natural processes and complicates understanding of the biogeographical history of species. This is exemplified by two invasive tunicates, *Ciona robusta* (formerly *Ciona intestinalis* type A) and *C. intestinalis* (formerly *Ciona intestinalis* type B), globally distributed and sympatric in Europe. By gathering new mitochondrial sequences that were merged with published datasets, we analysed genetic patterns in different regions, with a focus on 1) their sympatric range and 2) allopatric populations in N and S America and southern Europe. In the sympatric range, the two species display contrasting genetic diversity patterns, with low polymorphism in *C. robusta* supporting the prevalent view of its recent introduction. In the E Pacific, several genetic traits support the non-native status of *C. robusta.* However, in the NE Pacific, this appraisal requires a complex scenario of introduction and should be further examined supported by extensive sampling efforts in the NW Pacific (putative native range). For *C. intestinalis*, Bayesian analysis suggested a natural amphi-North Atlantic distribution, casting doubt on its non-native status in the NW Atlantic. This study shows that both natural and human-mediated dispersal have influenced genetic patterns at broad scales; this interaction lessens our ability to confidently ascertain native vs. non-native status of populations, particularly of those species that are globally distributed.

For centuries, species have been intentionally or accidentally transported beyond their native range by human activities (i.e. biological introduction^1^). Human-mediated dispersal has radically altered species’ distributions, sometimes leading to global distributions (i.e. with occupation of several biogeographic regions^2^). When introduction of a given taxon commenced in the relatively distant past, the identification of its native range can be difficult or even impossible, conferring cryptogenic status (uncertainty regarding native or non-native status^3^) over part or all of the species’ range. Several invertebrate species in the NW Atlantic that correspond to this category^4^,^5^,^6^ have been the subject of extensive debate in the scientific community^7^.

These issues are exemplified by the class Ascidiacea, in which more than 1600 species (out of 2815 valid species) were described only after 1950 and several have a worldwide distribution^8^. For instance, molecular and morphological studies have only recently confirmed *Ciona robusta* Hoshino and Tokioka, 1967 and *Ciona intestinalis* (Linnaeus, 1767) as distinct species. The two species were synonymized in 1985 under the name *Ciona intestinalis*^9^. However, in the early 2000s^10^,^11^,^12^, molecular studies reported four major evolutionarily divergent lineages in this species, among which were two types that were named *C. intestinalis* type A and type B. The taxonomic status of these two taxa has been recently re-evaluated: *C. intestinalis* type A was assigned to *C. robusta* described by Hoshino and Tokioka in 1967^13^ and *C. intestinalis* type B to *C. intestinalis* (Linnaeus, 1767), *sensu* Millar^14^. Several lines of evidence supported this re-classification: 1) morphological evidence showed distinctive features between the two taxa^15^,^16^,^17^, 2) the two taxa were first described in distinct biogeographic regions and oceans, in the North Pacific and North Atlantic for *C. robusta* and *C. intestinalis*, respectively, and 3) genetic and genomic studies showed their strong evolutionary divergence, estimated to have occurred ca. 3–5 My BP^12^,^18^,^19^.

The two species are distributed along temperate and warm-temperate coasts^20^,^21^ (Fig. 1). The larger range of *C. robusta* is, at least partly, explained by its tolerance to a larger temperature range compared to *C. intestinalis*^22^. The species are well established in artificial habitats including marinas and harbours, are important members of fouling communities and are both recognized as introduced/invasive species in parts of the world^23^,^24^.

*C. robusta* is generally assumed to be native to the NW Pacific, where it was described, and has been reported as an introduced species in the northern and southern hemisphere: in the Atlantic, Mediterranean Sea, Oceania, and North and South Pacific oceans (Fig. 1a, see the Supplementary Note for details). *C. intestinalis* is generally considered native to the NE Atlantic but non-native or cryptogenic in the NW Atlantic (e.g.^25^,^26^) and also occurs in the Bohai and Yellow Seas, China^21^ (Fig. 1b, Supplementary Note). The western English Channel and south of Brittany (hereafter named EC), is the only area confirmed so far where the two species live in sympatry, thought to result from the introduction of *C. robusta* into the native range of *C. intestinalis*^22^,^27^, although the Bohai and Yellow Seas are a second possible area^21^, as yet unconfirmed (Fig. 1b, Supplementary Note). In the EC contact zone, only extremely rare interspecific gene flow is occurring in the wild despite the two species living there in syntopy^22^,^27^: only a few hybrids were observed and all of them were shown to be first-generation (F1) hybrids; no other types of recent hybrid (e.g. backcrosses, F2 individuals) were observed so far^22^,^28^. Shared polymorphism was however documented and explained by gene flow during previous secondary contact between the two species^19^. According to Roux *et al.*^19^, this secondary contact occurred at a time estimated between 4–57 KY ago in an unknown location, before human activities substantially altered species’ natural ranges. Signs of this historical gene flow have indeed been reported in several allopatric regions in the Atlantic and Pacific^19^,^28^ suggesting a spread at a worldwide scale after the secondary contact, by natural expansion and/or human-mediated introduction.

Considering the re-classification of the two species, the recent report of new introductions (i.e. *C. robusta* in EC), the uncertainty of the native vs. native status in some regions (i.e. cryptogenic status of *C. intestinalis* in NW America) and their status as model species in evolutionary and developmental biology^20^, it is timely to describe global genetic patterns to better examine their histories and understand their contemporary distribution patterns and future on a global scale^29^.

Several studies have considered phylogenetic relationships within the genus *Ciona* or the *C. intestinalis* species complex, (e.g.^12^,^18^,^21^), or investigated genetic diversity and connectivity of local populations (e.g. in North America^25^, in South Africa^30^, and in Mediterranean Sea^31^). None of these examined in detail the global genetic patterns of the two species comparing different regions of introduction and notably the single sympatric area described so far. Here, we carried out genetic studies using sequence data obtained with two mitochondrial markers. Our data were merged with published information of Zhan *et al.*^25^ to produce population data at a global scale for the two species.

Besides providing a global picture, we had two more specific objectives. The first was to compare genetic patterns of the two species, which provide an almost perfectly matched set of biological properties (i.e. with shared phylogeny and life-history traits and very similar environmental requirements). They are both sessile as adults, with a short life cycle involving broadcast spawning for external fertilization, and with a non-feeding larva providing a short planktonic phase^20^,^22^,^32^. It has been argued^33^,^34^,^35^,^36^,^37^ that species’ basic biology will profoundly affect patterns of natural dispersal and thus population-genetic structuring and the extent of natural geographical ranges and rates of speciation. By this token, the two *Ciona* species should show very similar population-genetic properties. The extent to which they actually fulfil this expectation offers insight into the relative strength of biological characteristics vs. stochastic influences on dispersal and population demography, potentially including the intervention of anthropogenic vectors and major environmental events such as climatic fluctuations, which might by chance affect the species differently. Particularly close comparison of the species is possible in their contact zone in the EC, where the two species live side by side at many sites^22^.

Our second objective was to use the additional genetic information to assess the native vs. non-native status of the study species in the global regions sampled. The current understanding of the biogeographical status of the various populations is detailed, with references, in the Supplementary Note. In summary, *C. robusta* is presumed to be native to the NW Pacific, but a recent introduction in the English Channel (in the 2000s) and an older (19^th^ to mid-20^th^ century) introduction in the NE Pacific and Mediterranean Sea, while the age of the establishment in the SE Pacific is unclear. *C. intestinalis* is considered native to the NE Atlantic, while its status in the NW Atlantic is debated. Our analyses are based on patterns of genetic diversity and population structuring, with the assumption that both natural expansion and human-mediated contemporary dispersal could have influenced the observed patterns of genetic diversity. In the case of *C. intestinalis*, Bayesian analysis with an Isolation with Migration Model (IMM) is used to contrast alternative scenarios for the expansion of *C. intestinalis* in the North Atlantic.

## Results

**Figure 2:**
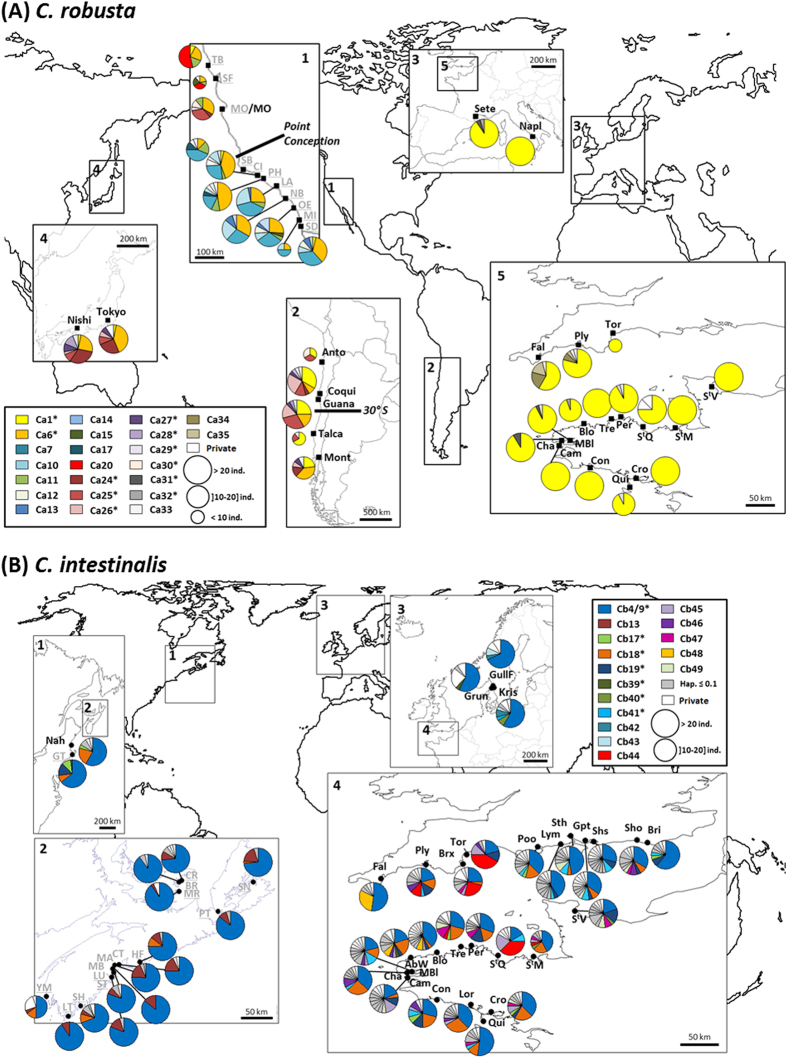
Location of the study populations and haplotype frequencies (COX3-ND1) at the population level for *C. robusta* (**A**) and *C. intestinalis* (**B**). Sites of *Ciona robusta* (**A**) and of *Ciona intestinalis* (**B**) sampled for this study are indicated in black and bold (for population codes and sampling, see Table S1). The localities studied by Zhan and co-authors^25^ that are included in the present study are in grey and underlined. Each color in pie charts refers to a given haplotype (see haplotype name in the box) as defined in the network in Fig. 3a[Fig f3]b for *C. robusta* and *C. intestinalis*, respectively. The haplotypes with a name followed by an asterisk are shared between at least two regions. For sake of clarity, only the haplotypes shared between regions or with frequency higher than 0.1 at population level are shown wth a specific color for *C. intestinalis*. Maps were obtained from http://www.pedagogie.ac-aix-marseille.fr/jcms/c_67064/fr/cartotheque under the permission of the creator (Daniel Dalet©Académie Aix-Marseille).

By merging our dataset with the published dataset of Zhan *et al.*^25^ on Pacific and Atlantic coasts of North America, totals of 714 *C. robusta* and 1140 *C. intestinalis* individuals were examined on COX3-ND1 sequences of 580 and 529 bp respectively. A subset of 501 individuals of *C. robusta* and 683 individuals of *C. intestinalis* sampled for this study (details about sampling are provided in Fig. 2 and Supplementary Table S1) were sequenced on an additional mtDNA fragment (COI). Concatenating CO1 and COX3-ND1 fragments allowed statistical analyses using a long fragment with a total of 1404 base pairs (bp) for *C. robusta* and 1313 bp for *C. intestinalis*. In the following text, for clarity, the results will be detailed for the largest sampling analyzed, thus with COX3-ND1; the results obtained with concatenated sequences are presented in the Supplementary Material and used in the main text only when relevant (e.g. to reinforce findings or point out contradictory results). Haplotype frequencies per population and sequences of haplotypes are deposited in DRYAD (DOI: 10.5061/dryad.7g555).

### Diversity analyses across species and populations

Genetic diversity indices obtained using our COX3-ND1 dataset with additional data from Zhan *et al.*^25^ are summarized for each region in Table 1 and detailed for each population in Supplementary Table S1. Haplotypic frequencies per population are illustrated in Fig. 2a,b for *C. robusta* and *C. intestinalis*, respectively. Very high polymorphism was observed over the two species but *C. robusta* was clearly less polymorphic than *C. intestinalis*, with 45 haplotypes (39 segregating sites) and 147 haplotypes (114 segregating sites) for *C. robusta* (N = 714) and *C. intestinalis* (N = 1140) respectively. As expected, the divergence was clearly higher between (14.4%) than within species (*C. robusta*: 0.71%, *C. intestinalis*: 1.96%).

**Figure 3 f3:**
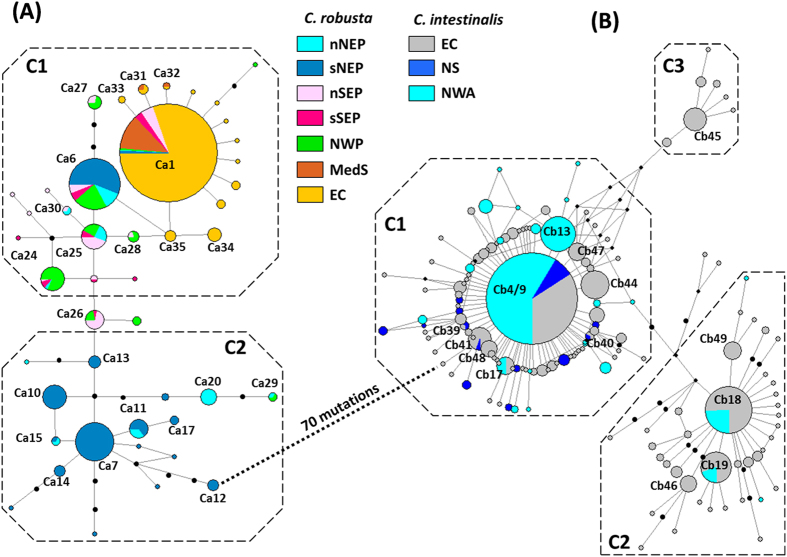
Median-joining haplotype networks of *Ciona robusta* (**A**) and *C. intestinalis* (**B**) based on COX3-ND1 sequences. Data from this study and from Zhan *et al.*^25^. Haplotype circles are proportional to haplotype frequency in the whole dataset. Branch lengths are proportional to number of mutational steps between two haplotypes. Missing haplotypes are indicated by small black circles. Colors represent the regions where the individuals possessing the haplotypes were found (regional codes are provided in Table 1).

Regarding genetic diversity indices for *C. robusta* at regional level, all Pacific areas sampled were similar (Hd = 0.792 for NW, Hd = 0.814 for NE and Hd = 0.749 for SE Pacific; Table 1). The same holds when considering mean values per population, for each region (Table 1). These values contrast with the very low genetic diversity found in EC at the regional (Hd = 0.138 for EC) and population (Hd_(mean)_ = 0.129 ± 0.153) level. These differences of population genetic diversity are significant between NE Pacific and EC (*P* < 0.001, Mann-Whitney rank sum test), SE Pacific and EC (*P* = 0.003) but not between the NE and SE Pacific ranges (*P* = 0.289). Similar statistical tests could not be reliably carried out with the NW Pacific as two populations only were examined in this region. Note that large differences were observed between the two Mediterranean populations studied (Hd_(mean)_ = 0.093 ± 0.132) indicating the need for further sampling in this region to investigate possible regional trends. The haplotypic richness, which makes correction for sampling effort, is also higher in Pacific ranges (9.0, 15.5 and 12.9 for NW, NE and SE Pacific, respectively) compared to EC (6.41) (Table 1). The low haplotypic diversity in European populations as compared to all other populations studied is well illustrated by the map in Fig. 2a; some populations were monomorphic (e.g. Cam, Con, StM for EC and Napl) despite the extensive polymorphism of the markers used. The nucleotide diversity over all populations of the EC was also very low with a π value 12 times lower than in the NW Pacific, 17 times lower than in the NE Pacific, and 10 times lower than in the SE Pacific. This is illustrated in the network (Fig. 3a) in which all the Europe haplotypes are closely clustered in a star-like feature (1 and 2 mutation steps) around a central dominant haplotype (Ca1). Conversely, Pacific haplotypes are distributed in the network in two clusters: C1 incorporated all individuals from the EC and Mediterranean Sea, 87% of the individuals of the NW Pacific, 88% of the SE Pacific and 37% of the NE Pacific; and C2 included the remaining individuals from the NE Pacific and 3% of the individuals of the NW Pacific (Supplementary Table S2a). Only private haplotype richness distinguished the three Pacific regions, with the highest values (4.46 and 5.20) for NE and SE Pacific whereas NW Pacific displayed a lower value (1.18) that was more similar to EC (1.67) (Table 1).

For *C. intestinalis*, the NE Atlantic showed almost twice the haplotypic diversity observed in the NW Atlantic (Hd = 0.854 for NE, Hd = 0.498 for NW, Table 1). Conversely to *C. robusta*, EC area was significantly more genetically diverse (Hd_(mean)_ = 0.839 ± 0.081) than elsewhere: the North Sea (Hd_(mean)_ = 0.608 ± 0.067, *P* < 0.01, Mann-Whitney rank sum test) and the NW Atlantic (Hd_(mean)_ = 0.482 ± 0.188, *P* < 0.001). However, Hd was not significantly different between North Sea and the NW Atlantic (*P* = 0.162). The high molecular diversity of the EC populations is illustrated by the haplotypic frequencies map (Fig. 2b) and the median joining haplotype network (Fig. 3b). EC haplotypes were distributed in three star-like clusters separated by 3–4 mutation steps (C1, C2 and C3, Supplementary Table S2b): Cluster C1 was represented by 67% of the EC individuals, C2 by 28% and C3 by 5%; for the NW Atlantic, haplotypes were represented by 94% of the individuals in C1 and 6% in C2 while haplotypes of the North Sea were all found in C1. Dominant haplotypes of the two major clusters (Cb4/9 for C1 and Cb18 of C2) were both shared by individuals of both Atlantic coasts. It is however important to note that most haplotypes were private to one coast (i.e. 84% and 94% of haplotypes were found only in NW and NE Atlantic respectively, Table 1).

The findings obtained with COX3-ND1 were all confirmed by using the concatenated sequences. In particular, *C. robusta* was clearly less polymorphic than *C. intestinalis*, with 48 haplotypes (63 segregating sites over 1404 bp) and 255 haplotypes (227 segregating sites over 1313 bp) for *C. robusta* and *C. intestinalis* respectively. Genetic diversity indices (Hd: haplotypic diversity and π: nucleotide diversity) are detailed in Supplementary Table S3. For *C. robusta*, as shown with COX3-ND1 only, the populations in the EC displayed the lowest haplotype diversity (mean per population, Hd_(mean)_ = 0.265 ± 0.232) compared to populations of other geographical areas, in particular SE Pacific populations (Hd_(mean)_ = 0.897 ± 0.066), where the highest population genetic diversity indices were found. This low molecular diversity in the EC is well illustrated by the haplotype network (Supplementary Figure S1) with a topology similar to the COX3-ND1 network (i.e. a star-like network with few haplotypes all at 1–3 mutation steps around a central dominant haplotype). The differences already observed between the two Mediterranean populations are even more pronounced with the concatenated dataset. For *C. intestinalis*, as with the COX3-ND1 locus alone, the genetic diversity was high in every population with an opposite trend to *C. robusta* in the EC: the EC contributed most of the diversity observed in *C. intestinalis*, as shown by the high haplotype diversity average Hd (0.960 ± 0.028) and explained by a large proportion of private haplotypes in this region. Compared to COX3-ND1, the concatenated dataset displayed more clearly the presence of three clusters (C1, C2 and C3 in Supplementary Figure S1b) with more divergence between them, i.e. separated by 11–12 mutations. Similarly to COX3-ND1, EC haplotypes were distributed in the three clusters, North Sea haplotypes were in only one cluster, NW Atlantic haplotype in the two major clusters (Supplementary Table S4), with for both, the sharing of central haplotypes by the two sides of the Atlantic.

### Genetic structure

**Figure 4:**
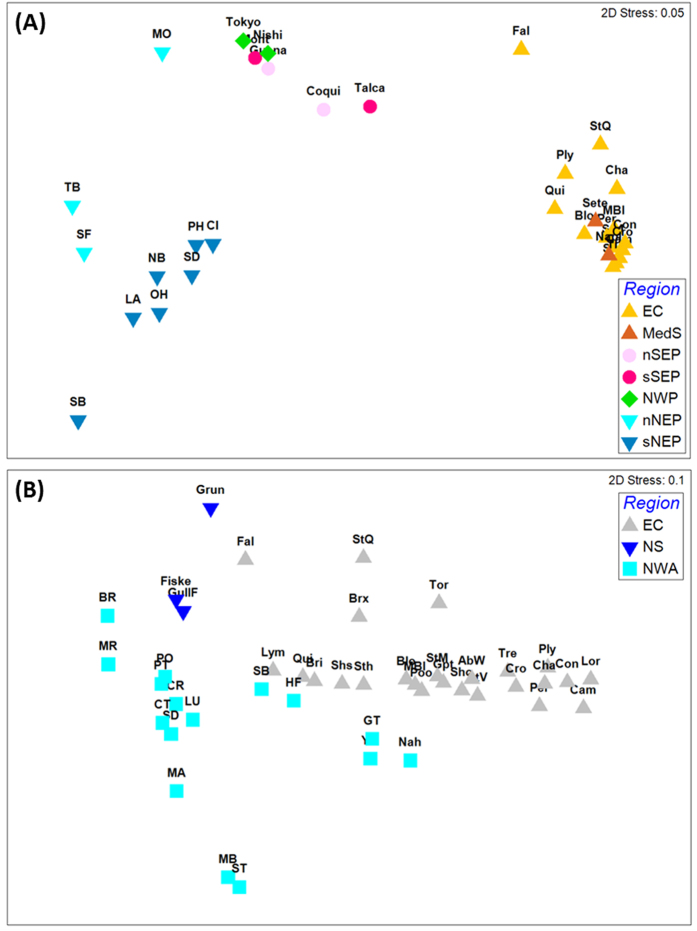
Non-metric multidimensional scaling plots constructed using pairwise F_ST_ estimates among (**A**) all populations of *C. robusta* and (**B**) all populations of *C. intestinalis*. The same colour code as in Fig. 3 was used to represent regions.

For *C. robusta*, overall genetic differentiation among all populations at COX3-ND1 locus was high and significant (ϕ_ST_ = 0.461, *P* < 10^−4^). The hierarchical AMOVA showed a large regional effect (ϕ_CT_ = 0.497, *P* < 10^−4^, Table 2) whereas genetic structure between localities within groups was low although significant (ϕ_SC_ = 0.083, *P* < 10^−4^, Table 2). Population pairwise ϕ_ST_ values (detailed in Supplementary Table S5a) are illustrated by the nMDS plot shown in Fig. 4a. The NW and SE Pacific regions appear genetically close. This genetic similarity is confirmed by a hierarchical AMOVA which showed an absence of significant effect of the regional grouping (ϕ_CT_ = 0.044, *P* = 0.075). The other regional groups clearly distinguished by the nMDS plot are also noticeable in the network (Fig. 3a) and the haplotype frequencies map (Fig. 2a). For instance, in the network, a single haplotype (Ca1) is shared by the five geographical areas but at high frequency in the EC and Mediterranean Sea (90% and 95% respectively), moderate frequency in the SE Pacific (33%) and low frequency in the remaining areas, the NW and NE Pacific (3.1 and 1.5% respectively). The NE Pacific, the only region for which haplotypes were found in the two clusters, is also a region with two sub-groups distinguished in the nMDS plot (northern and southern NE Pacific Fig. 4a). Interestingly these two groups are distributed across a well-known biogeographic break in the NE Pacific (i.e. at Point Conception)^38^. Hierarchical AMOVA carried out by grouping populations according to this biogeographic break showed a significant sub-group effect (ϕ_CT_ = 0.10, *P* < 10^−4^). A similar biogeographic break has been described in the SE Pacific at 30°S–33°S^39^, separating in our study the populations Anto, Coqui, Guana from Talca and Mont (Fig. 2a). Conversely to the NE Pacific, AMOVA did not show significant differences between these two groups in the SE Pacific (ϕ_CT_ = 0.020, *P = *0.198). In Europe, hierarchical AMOVA also did not show genetic differences between EC and Mediterranean Sea (ϕ_CT_ = 0.025, *P = *0.518).

For *C. intestinalis*, the overall genetic differentiation was significant among populations (ϕ_ST_) and the hierarchical AMOVA showed a significant effect of geographical areas (ϕ_CT_) and of populations within areas (ϕ_SC_) (Table 2). Excluding populations of North Sea from the AMOVA analysis did not change the results, still showing a significant effect of geographical area (ϕ_CT_ = 0.115, *P* < 10^−4^) and of populations within areas (ϕ_SC_ = 0.062, *P* < 10^−4^). Altogether, the outcome of the hierarchical genetic variance analyses is consistent with the network analysis showing no particular geographical partitioning of the three clusters (Fig. 3b). Pairwise genetic distances (Supplementary Table S5b) showed that the most differentiated populations in the NE Atlantic were those from the North Sea, as illustrated with the nMDS plot with the Grun population (Fig. 4b). The NW Atlantic populations appear genetically different from each other but with a subset (i.e. SB, HF, Nah, GT and YM, Fig. 4b) more similar to the EC populations than to other NW Atlantic populations.

Using the concatenated dataset on the subset of localities for which COI data were obtained confirm all the findings described with COX3-ND1 only. For example, for *C. robusta*, the hierarchical AMOVA confirmed a large regional effect (ϕ_CT_) (Table 2). Similarly, genetic similarities were observed between SE Pacific and NW Pacific: 33% of the haplotypes found in the SE and NW Pacific were shared between the two ranges. Also, at the European level, Naples in the Mediterranean Sea and populations of the EC were not genetically different as indicated by pairwise ϕ_ST_-values (Supplementary Table S6a). As for *C. robusta*, the concatenated dataset confirmed the findings detailed above for COX3-ND1 in *C. intestinalis* (data are detailed in Supplementary Tables S3b and S6b).

### Bayesian inference of divergence time for *C. intestinalis* in the N Atlantic

**Figure 5:**
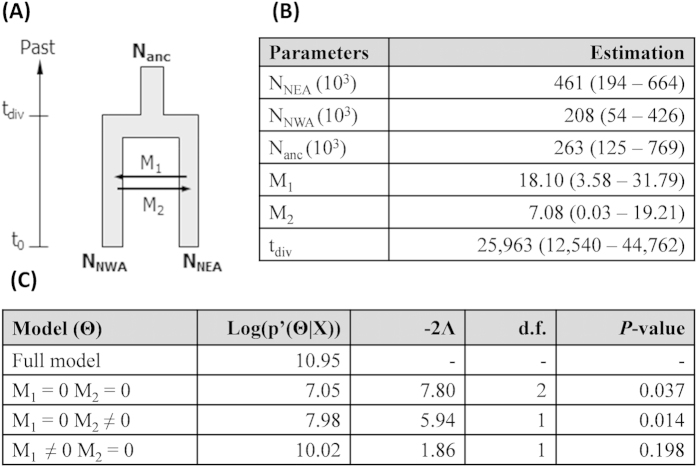
Demographic and divergence parameters estimated under isolation with migration model for *Ciona intestinalis*. (**A**) Diagram of the population splitting events (see Table 1 for codes of geographical areas). Time axis is not to scale. (**B**) Estimates of times of divergence and demographic parameters for the two studied regions using COX3-ND1 sequences. N_anc_, N_NEA_, N_NWA_: effective population size; M_1_ and M_2_: number of migrants per generation; t_div_: time since divergence. Median value and the 95% highest posterior density (95 HPD) of each parameter are given. (**C**) Tests for the migration model; Log(p’(Θ|X): log of the highest posterior density of each model; −2Λ: likelihood ratio; d.f.: degrees of freedom; and *P*-value.

Results of the Isolation with Migration Model (IMM) used for *C. intestinalis* to evaluate the time of divergence between NW Atlantic and NE Atlantic are provided in Fig. 5, showing the median value and the 95% highest posterior density (95HPD) of each parameter, and in Supplementary Figure S2, showing the marginal posterior distribution of each parameter. Under IMM, the time of divergence between NW Atlantic and NE Atlantic was estimated 25,963 yrs BP (95HPD: 12,540 – 44,762; Fig. 5b). Concerning the migration model, only the model with an asymmetric migration could not be rejected (Fig. 5c), suggesting substantial gene flow from NE to NW Atlantic but not from NW to NE Atlantic.

## Discussion

Whatever the dataset, contrasting patterns were observed in *Ciona robusta* and *C. intestinalis*. Altogether, *C. robusta* displayed lower genetic diversity at population and regional levels, but marked spatial genetic structure at these two scales. In contrast, *C. intestinalis* showed high genetic diversity and a more homogenous genetic structure in the N Atlantic. These contrasting patterns are even more pronounced in the sympatric area (i.e. EC): very low genetic diversity was observed in *C. robusta*, while *C. intestinalis* encompassed the whole genetic diversity observed at the species level in our study.

Roux and coauthors^19^ also recorded lower polymorphism in *C. robusta* compared to *C. intestinalis* at a species level. They analyzed few individuals and localities of the two species (i.e. 10 individuals sampled in 3 localities for the two species), but at the genome level (i.e. 852 nuclear loci isolated from full transcriptomes). We here confirm this global pattern with a mitochondrial marker, using a wider geographical coverage and more extensive population sampling, with the addition of three geographic regions: SE Pacific, NW Pacific and EC. mtDNA markers can be sensitive to selective effects^40^. However, results from our mitochondrial dataset were congruent with those obtained by Roux and coauthors^19^. In addition, a similar trend is apparent in a microsatellite study^25^ of *C. robusta* (type A) in the NE Pacific and *C. intestinalis* (type B) in the NW Atlantic: Mann-Whitney rank sum tests on data provided in Table 1 of Zhan *et al.*^25^ showed significant differences for gene diversity (*P* = 0.005) and allelic richness (*P* = 0.018), both lower in *C. robusta* as compared to *C. intestinalis*.

What can explain such a pervasive difference between two congeneric species that share common life-history traits and ecological properties? Roux and coauthors^19^ suggested that after their divergence and isolation (estimated ca. 3–5 My BP during the Pliocene^12^,^19^) but before their global spread, demographic expansion of *C. intestinalis* was more pronounced than in *C. robusta*, or that a stronger bottleneck occurred in *C.robusta* than in *C. intestinalis*. This last hypothesis is potentially realistic given several glacial and interglacial episodes since the divergence of the two species, which severely impacted the distribution ranges of other marine species^41^,^42^,^43^,^44^. Roux and coauthors^19^ noted that “*Tajima’s D was significantly more negative in C. intestinalis B* [C. intestinalis] *than in C. intestinalis A* [C. robusta]”. They however did not get statistical support for a demographic bottleneck based on various statistics (Tajima’s D, Fu and Li’s D and F), and hypothesized that this result might reflect a lack of statistical power, in particular stemming from insufficient sampling. Dedicated demographic studies, using nuclear markers and including a substantial sampling effort, notably in its Asian range, are needed to investigate in detail the cause of reduced genetic diversity in *C. robusta.* Decreased polymorphism of *C. robusta* as compared to *C. intestinalis* is nevertheless a consistent finding across the few studies carried out so far (this study and^19^,^25^) at different scales, in different regions and with various markers.

*C. robusta* is distributed across biogeographic provinces that are separated by natural barriers to dispersal (Fig. 2a). Our study revealed large differences in the level of genetic diversity across these regions. Based on the COX3-ND1 and concatenated datasets, we could divide the current range of *C. robusta* into two categories: i) regions of relatively high genetic diversity (all those sampled in the Pacific) and ii) one region of lower genetic diversity (EC). The Mediterranean Sea is not classified as only two populations displaying much contrasting features were available. Pacific populations are genetically distinct from European areas (Figs 2a and 4a). The presence of evolutionarily divergent and private haplotypes suggest long-term residence of *C. robusta* in Pacific regions^4^.

However, the NE Pacific shows distinctive features as compared to NW and SE Pacific regions, in particular the largest haplotypic diversity accompanied by the largest nucleotide diversity values (Table 1), thus highlighting the co-existence of particularly diverse and evolutionarily divergent haplotypes as pictured by the network analysis (Fig. 3a). In addition, except for the population MO (Monterey), NE Pacific populations are genetically distinct from the NW and SE Pacific, the two last-named regions being quite similar to each other as pictured by the nMDS plot (Fig. 4a). Finally, as shown by Zhan *et al.*^25^, individuals of the NE Pacific are distributed within two genetically differentiated groups located in the north and the south along the coasts (here after refered to nNEP and sNEP)

Can these genetic patterns cast light on the putative native (in the NW Pacific) and non-native or cryptogenic (in the NE and SE Pacific) status of *C. robusta* in the Pacific? Compared to the native range of a species, some genetic signs are expected in the non-native range, in particular: lower frequency or absence of private (endemic) haplotypes, weaker genetic structure, lower genetic diversity (except if the introduction was accompanied by genetic admixture between genetically differentiated sources) and lack of concordance between gene genealogies and the geographical distribution of the haplotypes. NE Pacific populations, showing high diversity and private haplotypes, do not fit particularly well with these characteristics. In addition an important genetic discontinuity separates populations located north and south of the well-known biogeographic break at Point Conception (Fig. 2a). This biogeographic boundary is a source of genetic differentiation for species showing a short pelagic phase^45^,^46^, like ascidians^32^. Based on these characteristics, we might hypothesize that *C. robusta* is native to the NE Pacific. However, these arguments are insufficient to dismiss the possible non-native status of *C. robusta* in this region. High levels of genetic diversity have in fact been reported in most non-native populations of marine invertebrates^29^, including non-native ascidians^47^,^48^. This is explained by propagule pressure due to the existence of multiple vectors (polyvectism *sensu* Carlton & Ruiz^49^) from genetically diverse sources^29^,^49^. Furthermore, not only the frequent haplotype Ca6 is shared by the two sub-regions nNEP and sNEP but also the less frequent haplotype Ca11 (Fig. 2a), and these two haplotypes belong to opposite clusters (C1 and C2 respectively) of the gene genealogy (Fig. 3a). NE Pacific populations thus display admixture between genetically divergent lineages, a pattern often observed in non-native species due to repeated introductions^50^. Such genetic mixing has already been described in the introduced range of *C. robusta* in South Africa^30^. In the NE Pacific, a non-native status for *C. robusta* is thus possible but the genetic characteristics observed involve 1) numerous and repeated introduction events from genetically diversified sources and 2) two distinct and independent introduction events, in the northern and southern part of this region, from at least two genetically differentiated sources, and subsequent gene flow between the two regions. This is not an exceptional scenario in marine invertebrates, as illustrated by the introduction of *Carcinus maenas* in the NW Atlantic which involved the establishment of two lineages from two independent introduction events^51^. So far, such a scenario is not strongly supported by the data obtained in NW Pacific: the two Japanese populations studied are genetically diverse but similar to each other (Fig. 4a) and only the haplotype Ca29 belongs to the C2 cluster common in the NE Pacific, and particularly in sNEP (Fig. 3a). An extensive sampling effort in the NW Pacific is thus needed to seek places where C2 haplotypes are frequent, and thus potential sources of the introductions to the NE Pacific.

By contrast with the NE Pacific, SE Pacific and NW Pacific populations were found to be poorly differentiated (Fig. 4a) and to share numerous haplotypes (Figs 2a and 3a), a situation involving very poor concordance between gene genealogy and geography. Given the great distance between them and their positions in opposite hemispheres, it is not considered feasible that both regions are within the native range of the species as part of an array of undifferentiated populations. Instead, it is presumed that one of these populations was derived from the other as a result of anthropogenic dispersal, apparently directly rather than through secondary introductions. Interestingly, the Chilean populations studied are spread over a recognized biogeographical boundary at 30–33°S: this boundary is known to be associated with a major phylogeographic break in low dispersers^39^,^52^, like *C. robusta*. And yet no genetic structure was found between populations located on both sides of this boundary (Anto, Coqui, Guana *versus* Talca, Mont). This finding supports the supposed non-native status of *C. robusta* in this region (Supplementary Note). Another interesting finding is that Chilean populations share one haplotype at high frequency with NE Atlantic populations (i.e. Ca1 haplotype in Figs 2a and 3a), suggesting that they might share the same origin or even that one region might have been seeded by the other. It is noteworthy that, despite their geographical remoteness, Europe hosts recently introduced species considered native to Chile, e.g. the tunicate *Corella eumyota*^53^ and the mollusc *Crepipatella dilatata*^54^; Chile and EC also share several non-native species, e.g. the tunicate *Asterocarpa humilis*, the bryozoan *Bugula neritina*, the mollusc *Mytilus galloprovincialis*, the green alga *Codium fragile fragile* (cited as *C. fragile tomentosoides* in Castilla *et al.*^55^) and the red alga *Polysiphonia morrowii*^56^,^57^,^58^.

The low diversity of the EC populations compared to Pacific populations appears consistent with a very recent introduction of *C. robusta* in this region. The EC populations did not display signs of genetic admixture (as might arise over time from multiple introductions) and most of them shared several haplotypes including the dominant Ca1 (Figs 2a and 3a) with the SE, NE and NW Pacific and the Mediterranean Sea. There is no overall concordance between gene genealogies and geography at this broad scale. Some haplotypes or branches of the haplotype network are shared across very distinct biogeographic regions. Such a pattern is incompatible with a hypothesis of natural expansion, particularly considering the poor dispersal ability of *C. robusta* and the broad geographical scale here considered^59^,^60^. The disjunct geographical distribution and weak genetic structure between the Mediterranean Sea and the NE Atlantic in *C. robusta* are also not expected within a native range^59^,^61^. Bottleneck events are rarely observed in marine invertebrates^29^ except in the very early stages of expansion. This scenario seems probable to be the situation for *C. robusta* in Europe and explain the low genetic diversity in this region. But besides this “selectively neutral” scenario, we need to consider “adaptive” scenarios for reduction in genetic diversity, for instance selection on standing genetic variation, which is one of the evolutionary outcomes of introductions of genetically diversified individuals^40^. A selective sweep in EC populations of *C. robusta* could explain our present findings based on mitochondrial loci^40^. However, 115 polymorphic SNPs examined in one population in Chile (Guanaqueros in the present study) also displayed higher genetic diversity (He = 0.294) than the corresponding markers in the EC populations (7 populations studied here, range of He = 0.236–0.253)^28^. It would be informative, as recently undertaken for other marine invaders^62^,^63^,^64^, to examine additional populations with nuclear markers and high genome coverage, not only to confirm patterns of lower genetic diversity in English Channel populations compared to the Pacific but also to examine in more detail the genetic diversity and structure of Pacific populations. Looking for the source of a European introduction was beyond the scope of this study as it would have required much more substantial sampling of the Asian range (e.g. *C. robusta* has also been reported in eastern Korea^65^) as well as of other regions (i.e. SW Pacific, S Atlantic) where the species is reported, and presumed to be introduced, representing possible sources for a secondary introduction into Europe.

Whereas our study agrees with the literature to support the non-native status of *C. robusta* in the NE Atlantic (Supplementary Note), it conversely casts doubt on the commonly assumed non-native status of *C. intestinalis* in NW Atlantic. In a previous study of patterns of marine species’ distributions in the N Atlantic, Haydar^7^ categorized *C. intestinalis* as possessing a disjunct distribution, being present on both E and W coasts of Atlantic but absent from the intervening Arctic coastal regions (i.e. Spitzbergen, Iceland, Greenland and northern Canada). The status of this species was accordingly classified as cryptogenic in NW Atlantic, its presence on both temperate coasts being attributable with certainty to neither natural nor anthropogenic dispersal. As an alternative to anthropogenic dispersal, on-going natural dispersal across the Atlantic seems unlikely for organisms such as *C. intestinalis* which have a brief planktonic phase^32^, but a naturally disjunct distribution could arise from recolonization of NW Atlantic coasts from glacial refuges, as has been suggested for the gastropod *Littorina littorea*^5^, a species alternatively regarded as a non-native in NW Atlantic^6^,^66^. Based on the COX3-ND1 dataset for *C. intestinalis*, the NW Atlantic population displays similarities to EC populations, with four haplotypes shared by populations on both N Atlantic coasts at medium and high frequencies, one being dominant in all populations (Cb4/9, Figs 2b and 3b). Additionally, weak regional genetic structure was found (Table 2). Disjunct distribution, shared dominant haplotypes and weak genetic structure are arguments in favor of an anthropogenic introduction of *C. intestinalis* across the Atlantic.

On the other hand, several lines of evidence in our study are consistent with the alternative hypothesis of *C. intestinalis* being native to both sides of the N Atlantic. A natural disjunct distribution occupying both sides of the N Atlantic has been ascribed to the ascidian *Cnemidocarpa mollis*^7^. In *Ciona intestinalis,* most of the haplotypes are private to the American (84%) or European (94%) region, and private haplotypes are often considered characteristic of long-term population establishment^4^,^5^. Also, the Bayesian inferences computed under IMM best supported the hypothesis of a natural divergence from a common ancestor (Fig. 5) within an estimated interval of 12,540 to 44,762 yrs BP. These dates not only exclude anthropogenic vectors but also bracket the Last Glacial Maximum (LGM; ca. 21ky BP) of the Pleistocene Period, a major driver of distribution range shifts^59^. In *C. intestinalis*, we observed high genetic diversity on both sides of the N Atlantic that was spread over the same two major haplotypic clusters in both mitochondrial datasets (Figs 4b and 5b). Such a partitioning is commonly explained by phylogeographic scenarios of divergence in allopatry followed by survival of lineages in different refugia and then by secondary contacts and gene flow during post-glacial expansion, as documented in the same region in several marine species^59^.

The three *Ciona intestinalis* populations of the North Sea (Skagerrak) showed a different pattern: they exhibited the lowest genetic diversity and were all included in the same haplotypic cluster. Such a pattern has already been described in several marine invertebrates and algae in the NE Atlantic (e.g. *Palmaria palmata*^67^; see Provan *et al.*^68^ for a review) and can reasonably be explained by a genetic bottleneck during retreat into a glacial refugium that was not balanced afterwards by massive expansion during post-glacial recolonization. Finally, it is noteworthy that *C. intestinalis* individuals have already been reported in the mid-20^th^ century in the Faeroe Islands^47^ and were more recently found in Iceland (SB, personal observation and sampling in 2014) suggesting that the species’ distribution is not as interrupted in the most northern areas of the N Atlantic as supposed. The present-day distribution of *C. intestinalis* might thus originate from natural expansion processes like those described for other coastal species, for instance *Littorina saxatilis*, characterized by an amphi-Atlantic distribution^69^ explained by its survival during glaciation period in multiple refugia on both sides of the N Atlantic. In this context, we note that *C. intestinalis* is largely replaced by two taxa of uncertain taxonomic rank along Arctic coasts, *C. gelatinosa* Bonnevie, 1896 and *C. longissim*a Hartmeyer, 1899. These were regarded as forms, varieties or subspecies of *Ciona intestinalis* by several authors, e.g.^9^,^70^,^71^,^72^,^73^, but were listed as full species by Sanamyan^74^. They have not been included in recent molecular clarifications of inter-relationships of Atlantic and global *Ciona* populations. If *C. gelatinosa* and *C. longissima* were found to be infraspecific entities within *C. intestinalis*, this species would have a continuous amphi-Atlantic distribution (see Fig. 8 in Dybern^75^), favouring the categorization of populations on both the E and W coasts as native according to the logical framework of Haydar^7^. Examining specimens preserved in museums and analyzing populations from natural habitats, rather than artificial ones, and from Arctic localities could be helpful to further assess the status of *C. intestinalis* in the NW Atlantic.

Note that natural isolation between the two populations does not exclude additional contemporary migrations. It is thus possible that both historical population splitting and human-mediated introductions have occurred in *Ciona intestinalis*, at different times, across the N Atlantic. This scenario is feasible given that some populations of the NW Atlantic are closely genetically related to populations in the NE Atlantic (e.g. Nah, GT, HF, Fig. 4b). This interaction can explain the observed genetic features and the ecological reports documenting a sudden expansion of *C. intestinalis* in the Maritime Provinces of Canada in recent decades^76^,^77^,^78^. It is also important to note that under IMM, mutation and migration rates are constant through time, an assumption rarely met in nature for migration, particularly for introduced species. More complex models assuming differential migration and mutation rates over time and using nuclear markers should be developed to evaluate more fully the demographic history of *C. intestinalis* in the N Atlantic. Despite these limitations, our data support a natural amphi-Atlantic distribution with plausible recent local population expansion due to human activities (i.e. human-mediated dispersal and increased coastal urbanization that opened new habitats to be colonized). As for the numerous cryptogenic species presenting similar complex patterns^7^, our study casts doubt on a single non-native origin for *C. intestinalis* in the NW Atlantic.

Despite the complexity of reconstructing the eco-evolutionary history of cosmopolitan and cryptogenic species that have possibly been exploiting human-made habitats and vectors for a long time, this study showed that mtDNA-based studies can be helpful in documenting contrasting features between congeners at a global scale. In the EC, although the two congeners display very similar biological features and occupy the same localities, they show contrasting genetic features, including notable differences in genetic diversity; this study highlighted the importance of population demographic events and human-mediated dispersal in determining genetic patterns, apparently over-riding the influence of life-history traits. Comparison between geographical regions revealed an apparent example in *C. robusta* in the NE Atlantic of reduced genetic diversity associated with recent introduction. This phenomenon is not commonly observed in marine invaders, presumably because the effects of founder events are short lived in the face of repeated introductions and high propagule loads. The low diversity observed in *C. robusta* in the EC is thus expected to disappear relatively quickly with new cryptic introductions, a hypothesis testable by temporal genetic monitoring. The data presented here also provide new insights regarding the native *vs.* non-native status of the two study species in various parts of their ranges. Our genetic data support a non-native status of *C. robusta* in the SE Pacific, particularly regarding the absence of genetic differences across the well-known biogeographic break at 30–33°S. However, our data do question the non-native status of *C. robusta* in the NE Pacific and of *C. intestinalis* in the NW Atlantic. Analyses of Arctic populations of *Ciona* sp. to assess the relationship of *C. intestinalis* populations on the two sides of the N Atlantic and additional studies in the NW Pacific range of *C. robusta* are priorities for further advancing the enquiries reported here. This study emphasizes the need for caution in the interpretation of global genetic patterns in species in which natural and human-mediated dispersal have interplayed for some time.

## Materials and Methods

### Sampling and DNA extraction

*Ciona robusta* and *C. intestinalis* populations were sampled in 25 sites of the EC (in most of which the two species were found living in syntopy, Fig. 2 and Supplementary Table S1 for details) and in 14 sites located in regions where only one of the two species has been reported so far (i.e. allopatric areas). In detail, *C. robusta* individuals were collected in 14 marinas in the EC, five locations in the SE Pacific (Chile), two in the putative native range, the NW Pacific (Japan), one in the NE Pacific and two in the Mediterranean Sea. Note that to our knowledge, this study is the first to sample a substantial number of individuals from the putative native range of *C. robusta* to carry out population genetic analyses. *C. intestinalis* individuals were collected in 25 marinas in the EC, three locations in the Skagerrak (North Sea region) and one in NW Atlantic (North America). Sampling was generally realized under floating pontoons, either using SCUBA diving or by collection from the surface from pontoon floats, hanging ropes and the underside of buoys. Around 24 individuals were sampled in each population with few exceptions (Supplementary Table S1 for details). For each individual, a piece of branchial basket was preserved in 100% ethanol for genetic analyses. DNA extraction was performed with a Nucleospin® 96 Tissue Kit (Macherey-Nagel, Germany) according to the manufacturer’s protocol.

### Sequencing procedure

Two mitochondrial loci (hereafter named mtDNA), cytochrome oxidase subunit 3 - NADH dehydrogenase subunit I (COX3-ND1) and cytochrome oxidase subunit I (COI), were chosen to allow comparisons with previous datasets^21^,^25^. The COX3-ND1 fragment was amplified using TX3F and TN1R primers following the PCR protocol described in Iannelli *et al.*^79^. The COI fragment was amplified using primer sequences and protocols obtained from Nydam and Harrison^12^. Sequencing reactions, run on an Applied BiosystemsTM AB3730XL DNA sequencer after purification on ExoSAP-it®, were performed at Centre National de Sequençage-Genoscope (Evry, France) or the LGC Genomics platform (Berlin, Germany). All PCR products were sequenced in both directions. Sequences obtained were edited using CodonCode Aligner v.4.0.2 (CodonCode Corporation, MA) and aligned using BioEdit v.7.1.9^80^. For COI, a final alignment length of 737 base pairs (bp) was obtained for each species. For COX3-ND1, the final alignment was 667 bp for *C. robusta* and 576 bp for *C. intestinalis*. Nucleotide sequences were translated into amino acid sequences using the Ascidian mitochondrial genetic code implemented in DnaSP v.5.10.01^81^.

### Statistical analyses

For a detailed comparison of the structure between Pacific and Atlantic coasts of North America^25^ and other regions of our sampling, the analyses were first carried out using COX3-ND1 only. We aligned our COX3-ND1 sequences and cut them to the same length as Zhan *et al.*^25^ (580 bp for *C. robusta* and 530 bp for *C. intestinalis*). Two overlapping peaks at position 434 bp for *C. intestinalis* were observed on the forward sequence but not on the reverse sequence. As we did not have access to the chromatographs obtained by Zhan and coauthors, we chose to exclude this position from the sequence analyses to avoid adding false haplotypes.

For each species and each population, the number of haplotypes (Nh), the number of polymorphic sites (S), the haplotype diversity (Hd) and the nucleotide diversity (π) were computed using DnaSP^81^. To compare diversity among populations and regions, haplotype richness and corrected number of private haplotypes were computed using the HP-RARE software^82^, which corrects for unequal sample sizes using a rarefaction procedure. For each species, median-joining networks were generated to infer the most parsimonious phylogenetic relationships among concatenated mtDNA haplotypes using NETWORK v.4.6 (www.fluxus-engineering.com). Analyses of genetic structure were carried out with ARLEQUIN v.3.5^83^. Population pairwise differentiations (ϕ_ST_) were carried out based on 10,000 random permutations. To picture the genetic distances between all study populations, a non-metric multi dimensional scaling (nMDS) was done with pairwise ϕ_ST_ values, using the PRIMER 6 + software^84^. To investigate genetic differences among (ϕ_CT_) and within (ϕ_SC_) geographic areas, a hierarchical analysis of molecular variance (AMOVA) was done.

The same analyses were also done using a subset of the sampling obtained for the two mitochondrial loci (i.e. all except the populations from Zhan *et al.*^21^,^25^) to check for the consistency of the results over a longer sequence. Due to the non-independence of the two loci, statistical analyses were carried out on concatenated sequences with a total of 1404 bp for *C. robusta* and 1313 bp for *C. intestinalis*. Note that similar results (in terms of genetic diversity and structure) were obtained independently when the two loci were analyzed separately (data not shown). Note that for AMOVA analyses for *C. intestinalis*, to avoid over-representation of the EC populations, the AMOVA was conducted with a random subsampling of 6 populations and was repeated with several other random subsamples of the populations without changing the results.

### Population divergence Bayesian analyses

Considering the cryptogenic status of *C. intestinalis*^7^, we investigated the divergence history between populations of this species present both sides of the N Atlantic. An Isolation with Migration Model (IMM) was performed using IMa2^85^. The IMM was applied to the COX3-ND1 dataset only, to include data from the NW Atlantic for *C. intestinalis*^25^. Briefly, the IMa2 program explores parameter and genealogy space through Markov chain Monte Carlo algorithms (MCMC) from a gene genealogy compatible with the observed dataset and generates posterior probabilities of several parameters. A Hasegawa-Kishino-Yano mutation model^86^ was used with a range of mutation rate between 1.31 × 10^−5^ and 2.67 × 10^−5^ per generation for the COX3-ND1 locus. These rates were estimated from the number of fixed mutations between *C. robusta* and *C. intestinalis* on COX3-ND1 sequences, divided by the lowest and the highest bound of the divergence time interval estimated to be 2.7–5.5 My BP, with one generation per year^19^. To optimize computation time, a subset of 80 individuals on each side of the N Atlantic was used (population splitting pictured in Fig. 5a). MCMC repetitions were done with 10^6^ steps after 100,000 long burn-in cycles. Finally, to test the influence of migration between areas after divergence, likelihood-ratio tests were performed on the nested model of gene flow according to Hey and Nielsen^87^. Note that the low sampling coverage of the putative Asian native range of *C. robusta* and the absence of sampling in the S Atlantic (i.e. South Africa and Brazil) and in SW Pacific (i.e. Australia and New Zealand) prevented us carrying out similar analyses for *C. robusta* in the Pacific.

## Additional Information

**How to cite this article**: Bouchemousse, S. *et al.* Contrasting global genetic patterns in two biologically similar, widespread and invasive *Ciona* species (Tunicata, Ascidiacea). *Sci. Rep.* **6**, 24875; doi: 10.1038/srep24875 (2016).

## Supplementary Material

Supplementary Information

## Figures and Tables

**Figure 1:**
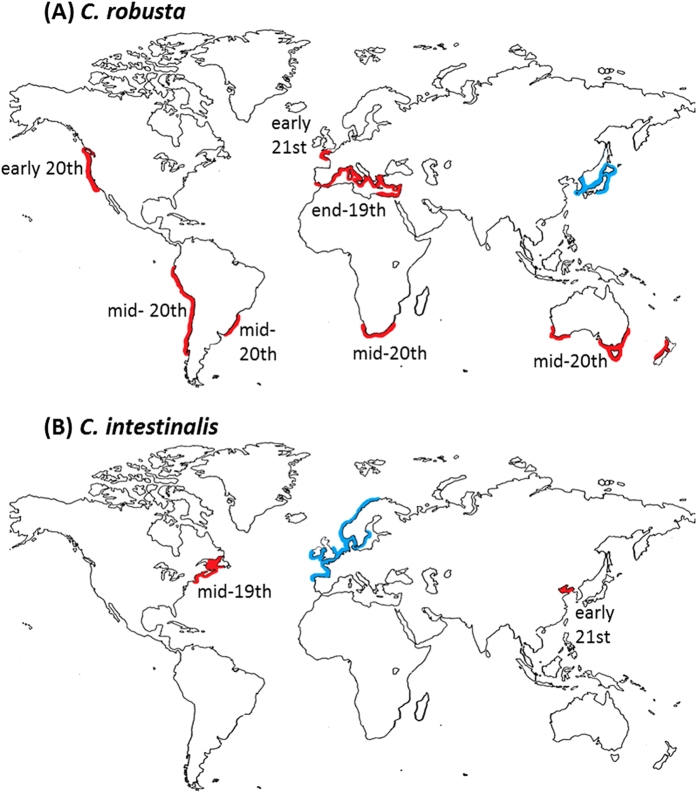
Worldwide distribution of *Ciona robusta* (**A**) and *C. intestinalis* (**B**) based on literature records. Areas where the species have been regarded as native or as non-native or cryptogenic (i.e. of undefined status) are in blue and red respectively. For each allopatric geographical region, the approximate time is given of the first report in the literature of *Ciona intestinalis sensu lato* (i.e. including both *C. intestinalis* and *C. robusta* before the distinction was appreciated); for the EC (sympatric region), the time shown for *C. robusta* is of its first documented presence as distinct from *C. intestinalis*; details in Supplementary Note. Map was obtained from http://www.pedagogie.ac-aix-marseille.fr/jcms/c_67064/fr/cartotheque under the permission of the creator (Daniel Dalet©Académie Aix-Marseille).

**Table 1:** Regional genetic diversity indices of *Ciona robusta* and *C. intestinalis* based on COX3-ND1 sequences (source: this study and Zhan *et al.* 25).

	Mean per population (±SD)									Total per region						
	Code	Npop	Nind	Nh	Rh	Npr	Rpr	Hd	π (10^2^)	Nind	Nh	Rh	Npr	Rpr	Hd	π (10^2^)
*C. robusta*																
North Western Pacific	NWP	2	32.00 (0.00)	6.50 (0.71)	6.06 (0.48)	0.50 (0.71)	0.73 (0.22)	0.790 (0.045)	0.301 (0.057)	64	9	8.98	1	1.18	0.792	0.301
North Eastern Pacific	NEP	10	20.50 (6.70)	6.20 (1.48)	5.33 (0.88)	0.80 (1.14)	0. 82 (0.84)	0.766 (0.050)	0.402 (0.098)	213	22	15.48	16	4.46	0.814	0.419
*-North NEP*	*nNEP*	*3*	*13.33 (4.51)*	*5.00 (1.00)*	*4.77 (0.78)*	*0.33 (0.58)*	*0.84 (0.73)*	*0.777 (0.092)*	*0.448 (0.162)*	*40*	*16*	*16.00*	*2*	*3.81*	*0.837*	*0.457*
*-South NEP*	*sNEP*	*7*	*23.57 (4.89)*	*6.71 (1.38)*	*5.56 (0.87)*	*1.00 (1.29)*	*0.81 (0.94)*	*0.775 (0.032)*	*0.382 (0.065)*	*173*	*9*	*5.51*	*12*	*3.35*	*0.775*	*0.393*
South Eastern Pacific	SEP	4	17.50 (7.68)	5.25 (2.06)	5.36 (1.66)	1.25 (0.50)	1.05 (0.28)	0.703 (0.111)	0.253 (0.024)	73	14	12.86	6	5.20	0.749	0.259
-*North SEP*	*nSEP*	*2*	*24.00 (0.00)*	*6.50 (2.12)*	*6.35 (0.76)*	*1.50 (0.71)*	*0.97 (0.42)*	*0.725 (0.056)*	*0.266 (0.031)*	*51*	*12*	*5.8*	*3*	*2.30*	*0.724*	*0.385*
-*South SEP*	*sSEP*	*2*	*11.00 (2.83)*	*4.00 (1.41)*	*4.37 (1.94)*	*1.00 (0.00)*	*1.13 (0.19)*	*0.682 (0.178)*	*0.241 (0.013)*	*22*	*8*	*8.0*	*2*	*2.00*	*0.771*	*0.246*
English Channel	EC	14	22.79 (3.31)	1.93 (1.00)	1.87 (0.97)	0.50 (0.76)	0.45 (0.59)	0.129 (0.153)	0.023 (0.028)	320	13	6.41	10	1.67	0.138	0.025
Mediterranean Sea	MedS	2	22.00 (1.41)	2.00 (1.41)	1.68 (0.96)	0.00 (0.00)	0.10 (0.14)	0.093 (0.132)	0.017 (0.023)	44	3	3	0	1.15	0.090	0.016
**Total**										**714**	**45**				**0.703**	**0.334**
*C. intestinalis*																
North Eastern Atlantic	NEA	28	23.54 (1.69)	10.68 (3.04)	10.40 (2.94)	2.89 (1.52)	2.81 (1.42)	0.815 (0.107)	0.732 (0.253)	659	126	44.45	119	21.19	0.854	0.815
-*English Channel*	*EC*	*25*	*23.68 (1.68)*	*11.24 (2.67)*	*10.93 (2.59)*	*3.00 (1.58)*	*2.90 (1.48)*	*0.839 (0.081)*	*0.793 (0.189)*	*592*	*117*	*43.36*	*110*	*20.79*	*0.870*	
* *-*North Sea*	*NS*	*3*	*22.33 (1.53)*	*6.00 (1.73)*	*5.93 (1.65)*	*2.00 (0.00)*	*2.04 (0.00)*	*0.608 (0.067)*	*0.223 (0.009)*	*67*	*13*	*13.00*	*9*	*6.19*	*0.619*	*0.232*
North Western Atlantic	NWA	16	30.06 (8.87)	4.81 (1.83)	4.14 (1.31)	0.88 (0.96)	0.83 (0.86)	0.482 (0.188)	0.241 (0.228)	481	25	13.47	21	9.81	0.498	0.324
**Total**										**1140**	**147**				**0.737**	**0.635**

For each region, per-population means and overall values for the region are given. Npop: number of populations; Nind: number of individuals; Nh: number of haplotypes; Rh: haplotypic richness (number of haplotypes corrected for sampling size); Npr: number of private haplotypes; Rpr: number of private haplotypes corrected for sampling size; Hd: haplotype diversity; π: nucleotide diversity. Genetic diversity indices per population are detailed in Supplementary Table S1. Study localities are shown in Fig. 2a for *C. robusta* and Fig. 2b for *C. intestinalis*.

**Table 2:** Hierarchical analysis of molecular variance for *Ciona robusta* and *C. intestinalis*.

	Fixation index	COX3-ND1 dataset		Concatenated dataset	
***C. robusta***	**Groups**	**NWP, NEP, SEP, EC and MedS**		**NWP, SEP, EC and MedS**	
	ϕ_CT_	0.497	(*P* < 0.001)	0.504	(*P* < 0.001)
	ϕ_SC_	0.083	(*P* < 0.001)	0.005	(*P* = 0.084)
	ϕ_ST_	0.539	(*P* < 0.001)	0.507	(*P* < 0.001)
***C. intestinalis***	**Groups**	**NWA, EC and NS**		**EC and NS**	
	ϕ_CT_	0.154	(*P* < 0.001)	0.125	(*P* < 0.001)
	ϕ_SC_	0.035	(*P* < 0.001)	0.071	(*P* < 0.001)
	ϕ_ST_	0.140	(*P* < 0.001)	0.147	(*P* < 0.001)

Analyses were done using the COX3-ND1 dataset and the concatenated mtDNA dataset. ϕ_CT_, ϕ_SC_ and ϕ_ST_ refer to the fixation index measuring genetic differences among areas, among populations within areas and among all populations, respectively. Probability values (H0: ϕ = 0) are indicated in brackets.
