# Supplementary material for: Contrasting global genetic patterns in two biologically similar, widespread and invasive Ciona species (Tunicata, Ascidiacea)
Source: Sci Rep. 2016 May 3;6:24875. doi: 10.1038/srep24875 (PMC4853746; doi:10.1038/srep24875)
Supplement: Supplementary Information [file srep24875-s1.pdf]

# Supplementary Material

## Contrasting global genetic patterns in two biologically similar, widespread and invasive *Ciona* species (Tunicata, Ascidiacea)

Sarah Bouchemousse[1*], John D.D. Bishop[2] and Frédérique Viard[1*]

**Supplementary Note. Taxonomic history of *C. robusta* and *C. intestinalis* and details of the historical observation of the two species, following literature report, in their introduced ranges at worldwide scale.**

Until September 2015, the nominal species *Ciona intestinalis* was considered a species complex that included four cryptic species named *C. intestinalis* type A to type D[1,2]. *C. intestinalis* type A and type B each have disjunct distributions and are considered as invasive species in several regions of the world[2]. During the period of absence of taxonomic assignation, the native ranges of these two types were debated[2-4]. However, recent alpha-taxonomic works[5,6] showed that *C. intestinalis* type A matched with the description of *C. robusta* Hoshino & Tokioka, 1967[7] (ecotype from Onagawa, Japan) and *C. intestinalis* type B with the description of *C. intestinalis* (Linnaeus, 1767) *sensu* Millar[8] (ecotype from Millport, Scotland). *C. robusta* had been placed synonymy with *C. intestinalis* by Hoshino & Nishikawa in 1985[9]. The classification of *C. intestinalis* type A and *C. intestinalis* type B as *C. robusta* and *C. intestinalis* has been accepted in WoRMS since September 2015. They are accepted as native to the region where they were described; the NW Pacific for *C. robusta* and the NE Atlantic for *C. intestinalis.*

*C. robusta* exhibits a disjunct global distribution in warm-temperate regions; this distribution and the fact that the species is most often restricted to urban habitats (ports, marinas) has been explained by introduction events in many regions, as listed below:

- English Channel: *C. robusta* (first reported as *C. intestinalis* type A) was first reported in the early 2000s in this range[10]. This species is well established along the eastern Brittany coastline[11], like several other ascidians recently introduced in this range, e.g. *Asterocarpa humilis*, *Corella eumyota*[12].
- Mediterranean Sea: Before the taxonomic re-evaluation in September 2015, the specimens were assigned to *C. intestinalis* and considered as members of a cryptogenic species[13,14]. The first report dates back to the end of the 19$^{th}$ century by Roule in the harbor of Marseille[15]. So far, *C. robusta* and other species of the genus *Ciona* (e.g. *C. edwardsi* and *C. roulei*) have been reported in the Mediterranean Sea but not *C. intestinalis sensu stricto*.
- SE Pacific: The first report of *C. robusta* (with the name *C. intestinalis* used until the recognition of *C. robusta* as a valid species) in this region is debated. Individuals classified as *C. intestinalis* were recorded in the Magellan Strait (Magellanic Province) in 1885 by Traustedt[16]. However, it is likely that this report correspond to *C. antarctica* Hartmeyer, 1911 and not *C. robusta*[17,18], considering that the Magellanic Province has a temperate-subantarctic biota whereas *C. robusta* lives in warm-temperate regions[19]. The next known report of *Ciona* was made by Van Name in 1949[20] in Antofagasta Bay (Peruvian Province; warm-temperate). More recent surveys[17] of ascidians carried out in the 2010s in the Magellan Strait and around Coquimbo (Peruvian Province) confirmed the absence of both *C. robusta* and *C. intestinalis* in the Magellanic Province and the presence of only *C. robusta* in the Peruvian Province. We can thus reasonably consider that the observation by Van

Name was the first report of *C. robusta* and that the presence of this species in Chile dates back (at least) to the mid-20th century.

- NE Pacific: Specimens reported under the name of *C. intestinalis* were first recorded in San Diego Bay in early part of the 20th century by Ritter & Forsyth[21] (cited by Lambert & Lambert[22]). Since the two species (or types) have been distinguished, only *C. robusta* (*C. intestinalis* type A) has been reported in this region. The species is considered to be non-native in this region [4,22-24].
- South Africa: *C. robusta* (first reported under the name of *C. intestinalis*) was reported for the first time in South Africa in the mid-20th century [25,26]. The species is identified as non-native in this region[27-29].
- Oceania: As in South Africa, *C. robusta* was reported under the name *C. intestinalis* in the mid-20th century in the Port Phillip (Victoria) in Australia by Millar[30]. The species is considered an invasive species in harbours of the southern coastline of Australia[31,32]. *C. robusta* was also reported in New Zealand (as *C. intestinalis*) during the second part of the 20th century[33].

Currently, *C. intestinalis* displays a disjunct distribution in the N Atlantic (i.e. reported in both E and W coasts but absent from Artic coastal regions) and it has been reported in one region outside the N Atlantic (see below).

- NW Atlantic: *C. intestinalis* was first reported in the Gulf of St Lawrence by Van Name[34]. The species is currently distributed from Rhode Island to Newfoundland[35] and is found at high density on artificial substrates along the south coast of Nova Scotia[36] and eastern coasts of Prince Edwards Island[37]. The recent proliferation of *C. intestinalis* in this region earned it the status of invasive species in most studies (e.g.[38-42]). The non-native status of *C. intestinalis* is however debated in this region (i.e. cryptogenic status) as for several other marine invertebrates presenting a similar distribution[43].
- NW Pacific: *C. intestinalis* (reported as *C. intestinalis* type B) was recorded very recently on the western coastline of Bohai Bay and Yellow Seas by Zhan et al.[2]. The lack of genetic differentiation between North American, European and Asian populations supported its classification as a non-native species in the NW Pacific. It is important to note that *C. robusta* (reported as *C. intestinalis* type A) has been reported in the East Sea and Korea Strait but not yet in the Yellow Sea[44] _ENREF_43.

## References


1 Nydam, M. L. & Harrison, R. G. Genealogical relationships within and among shallow-water *Ciona* species (Ascidiacea). *Mar. Biol.* **151**, 1839-1847; DOI:10.1007/s00227-007-0617-0 (2007).

2 Zhan, A., Macisaac, H. J. & Cristescu, M. E. Invasion genetics of the *Ciona intestinalis* species complex: from regional endemism to global homogeneity. *Mol. Ecol.* **19**, 4678-4694 (2010).

3 Caputi, L. *et al.* Cryptic speciation in a model invertebrate chordate. *Proc. Natl. Acad. Sci. of USA* **104**, 9364-9369; DOI:10.1073/pnas.0610158104 (2007).

4 Therriault, T. W. & Herborg, L.-M. Predicting the potential distribution of the vase tunicate *Ciona intestinalis* in Canadian waters: informing a risk assessment. *Ices J. Mar. Sci.* **65**, 788-794; DOI:10.1093/icesjms/fsn054 (2008).

5 Brunetti, R. *et al.* Morphological evidence that the molecularly determined *Ciona intestinalis type* A and type B are different species: *Ciona robusta* and *Ciona intestinalis*. *J. Zoolog. Syst. Evol. Res.* **53**, 186-193; DOI:10.1111/jzs.12101 (2015).

6 Pennati, R. *et al.* Morphological differences between larvae of the *Ciona intestinalis* species complex: Hints for a valid taxonomic definition of distinct species. *PloS ONE* **10**; DOI:10.1371/journal.pone.0122879 (2015).

7 Hoshino, Z.-i. & Tokiota, T. An unusually robust *Ciona* from the Northeastern coast of Honsyu Island, Japan. *Publ. Seto Mar. Biol. Lab.* **15**, 275-290 (1967).

8 Millar, R. H. in *L.M.B.C. Memoirs of typical british marine palnts and animals, XXXV* (ed J. S. Colman) 123 (Liverpool University Press, 1953).

9 Hoshino, Z.-i. & Nishikawa, T. Taxonomic Studies of *Ciona intestinalis* (L.) and its allies. *Publ. Seto Mar. Biol. Lab.* **30**, 61-79 (1985).

10 Bishop, J. D. D., Wood, C. A., Yunnie, A. L. E. & Griffiths, C. A. Unheralded arrivals: non-native sessile invertebrates in marinas on the English coast. *Aquat. Invasions* **10**, 249-264; DOI:10.3391/ai.2015.10.3.01 (2015).

11 Bouchemousse, S., Lévêque, L., Dubois, G. & Viard, F. Co-occurrence and reproductive synchrony do not ensure hybridization between an alien tunicate and its interfertile native congener. *Evol. Ecol.* **30**, 69-87; DOI:10.1007/s10682-015-9788-1 (2016).

12 Bishop, J. D. D., Wood, C. A., Leveque, L., Yunnie, A. L. E. & Viard, F. Repeated rapid assessment surveys reveal contrasting trends in occupancy of marinas by non-indigenous species on opposite sides of the western English Channel. *Mar. Pollut. Bull.* **95**, 699-706; DOI:10.1016/j.marpolbul.2014.11.043 (2015).

13 Airoldi, L., Turon, X., Perkol-Finkel, S. & Rius, M. Corridors for aliens but not for natives: effects of marine urban sprawl at a regional scale. *Divers. Distrib.* **21**, 755-768; DOI:10.1111/ddi.12301 (2015).

14 Lopez-Legentil, S., Legentil, M. L., Erwin, P. M. & Turon, X. Harbor networks as introduction gateways: contrasting distribution patterns of native and introduced ascidians. *Biol. Invasions* **17**, 1623-1638; DOI:10.1007/s10530-014-0821-z (2015).

15 Roule, L. in *Annales du musée d'histoire naturelle de Marseille* (ed A.F. Marion) (Typographie et Lithofraphie J. Cayer, 1884).

16 Traustedt, M. P. A. Ascidiae simplices fra det Stille Ocean Vidensk. 1-160 (Foren, Kjobenhavn, 1885).

17 Turon, X., Canete, J. I., Sellanes, J., Rocha, R. M. & Lopez-Legentil, S. Too cold for invasions? Contrasting patterns of native and introduced ascidians in subantarctic and temperate Chile. *Manag. Biol. Invasions* **in press** (2016).

18 Monniot, C. & Monniot, F. Ascidies antarctiques et subantarctiques: morphologie et biogéographie. *Mémoires du Muséum National d'Histoire Naturelle, Paris, série A, Zoologie* **125**, 1-180 (1983).

19 Procaccini, G., Affinito, O., Toscano, F. & Sordino, P. in *Evolutionary Biology: Concepts, Biodiversity, Macroevolution and Genome Evolution* (ed P. Pontarotti) Ch. 6, 91-106 (2011).

20 Van Name, W. G. Reports of the Lund University Chile expedition 1948-1949: Ascidians (Ascidiacea). (Lund Universitets Arsskrift, Lund, 1954).

21 Ritter, W. E. & Forsyth, R. A. Ascidians of the littoral zone of southern California. *University of California publications in zoology* **16**, 439-512 (1917).

22 Lambert, C. C. & Lambert, G. Non-indigenous ascidians in southern California harbors and marinas. *Mar. Biol.* **130**, 675-688 (1998).

23 Rius, M., Potter, E. E., Aguirre, J. D. & Stachowicz, J. J. Mechanisms of biotic resistance across complex life cycles. *J. Anim. Ecol.* **83**, 296-305; DOI:10.1111/1365-2656.12129 (2014).

24 Blum, J. C. *et al.* The non-native solitary ascidian *Ciona intestinalis* (L.) depresses species richness. *J. Exp. Mar. Biol. Ecol.* **342**, 5-14; DOI:10.1016/j.jembe.2006.10.010 (2007).

25 Millar, R. H. On the collection of ascidians from South Africa. *Proc. R. Soc. B* **125**, 169-221 (1955).

26 Michaelsen, W. The ascidians of the Cape Province of South Africa. *T. Roy. Soc. S. Afr.* **22**, 129-167 (1934).

27 Rius, M. *et al.* Range expansions across ecoregions: interactions of climate change , physiology and genetic diversity. *Global Ecol. Biogeogr.* **23**, 76-88; DOI:10.1111/geb.12105 (2014).

28 Rius, M., Heasman, K. G. & McQuaid, C. D. Long-term coexistence of non-indigenous species in aquaculture facilities. *Mar. Pollut. Bull.* **62**, 2395-2403; DOI:10.1016/j.marpolbul.2011.08.030 (2011).

29 Robinson, T. B., Griffiths, C. L., McQuaid, C. D. & Rius, M. Marine alien species of South Africa: status and impacts. *Afr. J. Mar. Sci.* **27**, 297-306 (2005).

30 Millar, R. H. Ascidiacea, Port Phillip Survey 1957-1963. *Memoirs of the National Museum of Victoria (Melbourne)* **27**, 357-375 (1966).

31 MacDonald, J. The invasive pest species *Ciona intestinalis* (Linnaeus, 1767) reported in a harbour in southern Western Australia. *Mar. Pollut. Bull.* **49**, 868-870 (2004).

32 Hewitt, C. L. *et al.* Introduced and cryptogenic species in Port Phillip Bay, Victoria, Australia. *Mar. Biol.* **144**, 183-202; DOI:10.1007/s00227-003-1173-x (2004).

33 Millar, R. H. The marine fauna of New Zealand: Ascidiacea. *New Zealand Institut Memories* **85**, 117 p. (1982).

34 Van Name, W. G. The North and South American ascidians. *B. Am. Mus. Nat. Hist.* **84**, 1-476 pls. 471-431 (1945).

35 Sargent, P. S., Wells, T., Matheson, K., McKenzie, C. H. & Deibel, D. First record of vase tunicate, *Ciona intestinalis* (Linnaeus, 1767), in coastal Newfoundland waters. *BioInvasions Rec.* **2**, 89-98; DOI:10.3391/bir.2013.2.2.01 (2013).

36 Cayer, D., MacNeil, N. & Bagnall, A. G. Tunicate fouling in Nova Scotia aquaculture: a new development. *J. Shellfish Res.* **18**, 327 (1999).

37 Locke, A., Hanson, J. M., Ellis, K. M., Thompson, J. & Rochette, R. Invasion of the southern Gulf of St. Lawrence by the cludded tunicate (*Styela clava* Herdman): Potential mechanisms for invasions of Prince Edwards Island estuaries. *J. Exp. Mar. Biol. Ecol.* **342**, 69-77; DOI: 10.1016/j.jembe.2006.10.016 (2007).

38 Zhan, A. *et al.* Scale-dependent post-establishment spread and genetic diversity in an invading mollusc in South America. *Divers. Distrib.* **18**, 1042-1055; DOI:10.1111/j.1472-4642.2012.00894.x (2012).

39 Ramsay, A., Davidson, J., Bourque, D. & Stryhn, H. Recruitment patterns and population development of the invasive ascidian *Ciona intestinalis* in Prince Edward Island, Canada. *Aquat. Invasions* **4**, 169-176; DOI:10.3391/ai.2009.4.1.17 (2009).

40 Ramsay, A., Davidson, J., Landry, T. & Arsenault, G. Process of invasiveness among exotic tunicates in Prince Edward Island, Canada. *Biol. Invasions* **10**, 1311-1316; DOI:10.1007/s10530-007-9205-y (2008).

41 Vercaemer, B., Sephton, D., Nicolas, J. M., Howes, S. & Keays, J. *Ciona intestinalis* environmental control points: field and laboratory investigations. *Aquat. Invasions* **6**, 477-490; DOI:10.3391/ai.2011.6.4.13 (2011).

42 Collin, S. B., Edwards, P. K., Leung, B. & Johnson, L. E. Optimizing early detection of non-indigenous species: Estimating the scale of dispaersal of a nascent population of the invasive tunicate *Ciona intestinalis* (L.). *Mar. Pollut. Bull.* **73**, 64-69; DOI: 10.1016/j.marpolbul.2013.05.040 (2013).

43 Haydar, D. What is natural? The scale of cryptogenesis in the North Atlantic Ocean. *Divers. Distrib.* **18**, 101-110; DOI:10.1111/j.1472-4642.2011.00863.x (2012).

44 Lee, T. & Shin, S. Morphological and molecular identification of an introduced aline sea squirt (Tunicata: Ascidiacea) in Korea. *P. Biol. Soc. Wash.* **127**, 284-297; DOI:10.2988/0006-324X-127.1.284 (2014).

**Table S1. Sampling locations and details of genetic diversity indices of *Ciona robusta* and *C. intestinalis* computed from the mitochondrial COX3-ND1 sequences dataset** (source: this study and Zhan et al.[25]).

The English Channel is the only sympatric region wherein the two species were reported. S: syntopic localities, i.e. wherein the two species coexist in the same habitat; - : localities where *C. robusta* has never been reported so far (most recent surveys in autumn 2014, JDDB, pers. obs.).

Nind: the number of individuals studied; nf: not found. Indices: Nh: number of haplotypes; Rh: haplotypic richness, with rarefaction size within brackets; Npr: number of private haplotypes; Rpr: corrected number (for sampling size) of private haplotype; S: number of polymorphic sites; Hd: Haplotype diversity; $\pi$: nucleotide diversity.

| | | | ***C. robusta*** | | | | | | | | ***C. intestinalis*** | | | | | | | | **Source** |
|---|---|---|---|---|---|---|---|---|---|---|---|---|---|---|---|---|---|---|---|
| **Sampling location** | **Code** | **Status** | **Nind** | **Nh** | **Rh[9]** | **Npr** | **Rpr[9]** | **S** | **Hd** | **$\pi$ ($10^2$)** | **Nind** | **Nh** | **Nh[19]** | **Npr** | **Npr[19]** | **S** | **Hd** | **$\pi$ ($10^2$)** | |
| North Eastern Atlantic | NEA | | | | | | | | | | | | | | | | | | |
| *English Channel* | EC | | | | | | | | | | | | | | | | | | |
| Brighton, UK | Bri | - | nf | | | | | | | | 23 | 9 | 8.8 | 2 | 2.0 | 17 | 0.636 | 0.519 | |
| Shoreham, UK | Sho | - | nf | | | | | | | | 24 | 13 | 12.7 | 3 | 3.0 | 21 | 0.884 | 0.892 | |
| Southsea, UK | Shs | - | nf | | | | | | | | 24 | 16 | 15.5 | 6 | 5.9 | 24 | 0.917 | 0.719 | |
| Gosport, UK | Gpt | - | nf | | | | | | | | 23 | 14 | 13.7 | 6 | 5.9 | 23 | 0.881 | 0.932 | |
| Southampton, UK | Sth | - | nf | | | | | | | | 21 | 10 | 10.0 | 2 | 2.1 | 17 | 0.776 | 0.672 | |
| Lymington, UK | Lym | - | nf | | | | | | | | 22 | 13 | 12.8 | 3 | 3.0 | 18 | 0.840 | 0.466 | |
| Poole Quay, UK | Poo | - | nf | | | | | | | | 24 | 12 | 11.6 | 4 | 3.9 | 20 | 0.844 | 0.789 | |
| Torquay, UK | Tor | S | 1 | 1 | - | 0 | - | - | - | - | 24 | 7 | 6.9 | 1 | 1.0 | 15 | 0.779 | 0.839 | |
| Brixham, UK | Brx | - | nf | | | | | | | | 24 | 11 | 10.7 | 3 | 2.9 | 17 | 0.877 | 0.740 | |
| Plymouth, UK | Ply | S | 24 | 4 | 3.7 | 2 | 1.2 | 3 | 0.308 | 0.056 | 24 | 12 | 11.8 | 3 | 2.9 | 20 | 0.924 | 0.924 | |
| Falmouth, UK | Fal | S | 24 | 3 | 3.6 | 1 | 1.1 | 2 | 0.409 | 0.074 | 23 | 6 | 5.9 | 2 | 2.0 | 12 | 0.656 | 0.265 | |
| Saint Vaast, Fr | StV | S | 24 | 1 | 1.0 | 0 | 0.0 | 0 | 0.000 | 0.000 | 25 | 14 | 13.5 | 4 | 3.8 | 25 | 0.930 | 0.913 | |
| Saint Malo, Fr | StM | S | 23 | 1 | 1.0 | 0 | 0.0 | 0 | 0.000 | 0.000 | 19 | 9 | 9.0 | 1 | 1.0 | 14 | 0.801 | 0.745 | This study |
| Saint Quay, Fr | StQ | S | 24 | 3 | 2.9 | 2 | 1.9 | 2 | 0.424 | 0.079 | 24 | 8 | 7.8 | 4 | 3.6 | 17 | 0.808 | 0.689 | |
| Perros Guirec, Fr | Per | S | 24 | 3 | 2.9 | 1 | 0.7 | 2 | 0.163 | 0.029 | 26 | 11 | 10.6 | 2 | 2.0 | 20 | 0.871 | 0.959 | |
| Trébeurden, Fr | Tre | S | 24 | 1 | 1.0 | 0 | 0.0 | 0 | 0.000 | 0.000 | 24 | 10 | 9.8 | 2 | 2.0 | 18 | 0.870 | 0.974 | |
| Roscoff-Bloscon, Fr | Blo | S | 18 | 2 | 2.3 | 0 | 0.2 | 1 | 0.111 | 0.019 | 26 | 16 | 15.0 | 7 | 6.5 | 28 | 0.858 | 0.900 | |
| Aber Wrac'h, Fr | AbW | - | nf | | | | | | | | 25 | 13 | 12.6 | 2 | 2.0 | 22 | 0.920 | 0.946 | |
| Brest-Château, Fr | Cha | S | 23 | 2 | 1.7 | 0 | 0.0 | 1 | 0.166 | 0.029 | 27 | 10 | 9.4 | 2 | 1.9 | 16 | 0.815 | 0.930 | |
| Brest-Moulin Blanc, Fr | MBl | S | 26 | 2 | 1.5 | 1 | 0.6 | 1 | 0.077 | 0.013 | 24 | 13 | 12.8 | 5 | 4.1 | 23 | 0.932 | 0.885 | |
| Camaret, Fr | Cam | S | 24 | 1 | 1.0 | 0 | 0.0 | 0 | 0.000 | 0.000 | 21 | 15 | 14.9 | 3 | 3.1 | 27 | 0.952 | 0.956 | |
| Concarneau, Fr | Con | S | 24 | 1 | 1.0 | 0 | 0.0 | 0 | 0.000 | 0.000 | 24 | 9 | 8.9 | 2 | 1.9 | 16 | 0.866 | 0.913 | |
| Lorient, Fr | Lor | - | nf | | | | | | | | 24 | 10 | 9.7 | 2 | 2.0 | 19 | 0.793 | 0.877 | |
| Crouesty, Fr | Cro | S | 24 | 1 | 1.0 | 0 | 0.0 | 0 | 0.000 | 0.000 | 24 | 11 | 10.7 | 2 | 1.9 | 19 | 0.830 | 0.923 | |
| Quiberon, Fr | Qui | S | 13 | 2 | 1.9 | 0 | 0.4 | 1 | 0.154 | 0.027 | 23 | 9 | 8.8 | 2 | 2.0 | 15 | 0.723 | 0.460 | |
| *Total EC* | | | *320* | *13* | | *10* | | *10* | *0.138* | *0.025* | *592* | *117* | | *110* | | *100* | *0.870* | *0.854* | |
| *North Sea (Skagerrak)* | NS | | | | | | | | | | | | | | | | | | |
| Grundsund, Sw | Grun | | | | | | | | | | 21 | 5 | 5.0 | 2 | 2.0 | 5 | 0.633 | 0.214 | This study |
| Gullmar Fjord, Sw | GullF | | | | | | | | | | 22 | 5 | 5.0 | 2 | 2.0 | 8 | 0.532 | 0.231 | |

| | | | | | | | | | | | | | | | | | | |
|---|---|---|---|---|---|---|---|---|---|---|---|---|---|---|---|---|---|---|
| Fiskebäckskil, Sw | Fiske | | | | | | | | | 24 | 8 | 7.8 | 2 | 2.0 | 7 | 0.659 | 0.224 | |
| *Total NS* | | | | | | | | | | *67* | *13* | | *9* | | *13* | *0.619* | *0.232* | |
| *Total NEA* | | *320* | *13* | | *10* | | *10* | *0.138* | *0.025* | *659* | *126* | | *122* | | *103* | *0.854* | *0.815* | |
| North Western Atlantic | NWA | | | | | | | | | | | | | | | | | |
| Cardigan River, Ca | CR | | | | | | | | | 30 | 8 | 5.6 | 3 | 2.6 | 7 | 0.556 | 0.188 | |
| Brudenell River, Ca | BR | | | | | | | | | 30 | 3 | 2.9 | 1 | 0.9 | 2 | 0.191 | 0.037 | |
| Murray River, Ca | MR | | | | | | | | | 30 | 3 | 2.9 | 1 | 1.0 | 2 | 0.191 | 0.037 | |
| Sydney, Ca | SD | | | | | | | | | 42 | 5 | 3.6 | 0 | 0.2 | 9 | 0.577 | 0.195 | |
| Point Tupper, Ca | PO | | | | | | | | | 21 | 3 | 3.0 | 0 | 0.0 | 2 | 0.267 | 0.052 | |
| Halifax, Ca | HF | | | | | | | | | 28 | 4 | 3.9 | 0 | 0.0 | 7 | 0.429 | 0.308 | |
| Chester, Ca | CT | | | | | | | | | 28 | 4 | 3.8 | 2 | 1.8 | 5 | 0.418 | 0.124 | [25] |
| Martin's River, Ca | MA | | | | | | | | | 45 | 5 | 4.6 | 1 | 0.7 | 8 | 0.574 | 0.146 | |
| Mahone Bay, Ca | MB | | | | | | | | | 28 | 3 | 3.0 | 0 | 0.0 | 5 | 0.569 | 0.121 | |
| Stone Hurst, Ca | ST | | | | | | | | | 26 | 6 | 4.9 | 1 | 1.0 | 7 | 0.652 | 0.171 | |
| Lunenburg, Ca | LU | | | | | | | | | 21 | 3 | 3.0 | 0 | 0.1 | 3 | 0.338 | 0.079 | |
| Shelburne, Ca | SB | | | | | | | | | 39 | 7 | 5.6 | 1 | 1.0 | 13 | 0.547 | 0.386 | |
| Port La Tour, Ca | PT | | | | | | | | | 21 | 3 | 2.0 | 0 | 0.0 | 2 | 0.267 | 0.052 | |
| Yarmouth, Ca | YM | | | | | | | | | 20 | 8 | 6.0 | 2 | 2.0 | 14 | 0.821 | 0.619 | |
| Nahant, US | Nah | | | | | | | | | 24 | 6 | 5.9 | 2 | 1.9 | 16 | 0.630 | 0.740 | This study |
| Groton, US | GT | | | | | | | | | 48 | 6 | 5.6 | 0 | 0.0 | 12 | 0.688 | 0.607 | [25] |
| *Total NWA* | | | | | | | | | | *481* | *25* | | *20* | | *31* | *0.498* | *0.324* | |
| Mediterranean Sea | MedS | | | | | | | | | | | | | | | | | |
| Naples, It | Napl | 23 | 1 | 1.0 | 0 | 0.0 | 0 | 0.000 | 0.000 | | | | | | | | | This study |
| Sete, Fr | Sete | 21 | 3 | 2.4 | 0 | 0.2 | 2 | 0.186 | 0.033 | | | | | | | | | |
| *Total MedS* | | *44* | *3* | | *0* | | *2* | *0.090* | *0.016* | | | | | | | | | |
| North Western Pacific | NWP | | | | | | | | | | | | | | | | | |
| Nishinomiya, Japan | Nishi | 32 | 7 | 6.4 | 0 | 0.6 | 8 | 0.821 | 0.341 | | | | | | | | | This study |
| Tokyo, Japan | Tokyo | 32 | 6 | 5.7 | 1 | 0.9 | 7 | 0.758 | 0.261 | | | | | | | | | |
| *Total NWP* | | *64* | *9* | | *1* | | *9* | *0.792* | *0.301* | | | | | | | | | |
| North Eastern Pacific | NEP | | | | | | | | | | | | | | | | | |
| north NEP | nNEP | | | | | | | | | | | | | | | | | |
| Tomales Bay, US | TB | 13 | 4 | 3.9 | 0 | 0.0 | 7 | 0.679 | 0.471 | | | | | | | | | [25] |
| San Francisco Estuary, US | SF | 9 | 5 | 5.0 | 1 | 1.0 | 9 | 0.861 | 0.597 | | | | | | | | | [25]+This study |
| Monterey Bay, US | MO | 18 | 6 | 5.4 | 0 | 1.1 | 7 | 0.791 | 0.275 | | | | | | | | | [25] |
| *Total nNEP* | | *40* | *9* | | *2* | | *10* | *0.837* | *0.457* | | | | | | | | | |
| south NEP | sNEP | | | | | | | | | | | | | | | | | |
| Santa Barbara, US | SB | 16 | 6 | 5.6 | 2 | 1.9 | 6 | 0.783 | 0.298 | | | | | | | | | |
| Channel Islands, US | CI | 27 | 8 | 6.0 | 2 | 1.4 | 12 | 0.772 | 0.434 | | | | | | | | | |
| Port Hueneme, US | PH | 21 | 8 | 6.3 | 3 | 2.1 | 13 | 0.752 | 0.465 | | | | | | | | | |
| Los Angeles, US | LA | 31 | 5 | 4.3 | 0 | 0.0 | 5 | 0.735 | 0.321 | | | | | | | | | [25] |
| Newport Bay, US | NB | 21 | 5 | 4.6 | 0 | 0.0 | 5 | 0.776 | 0.333 | | | | | | | | | |
| Oceanside Estuary, US | OE | 23 | 8 | 4.6 | 0 | 0.2 | 9 | 0.838 | 0.429 | | | | | | | | | |
| Mission Bay, US | MI | 8 | 3 | - | 0 | | 4 | 0.714 | 0.322 | | | | | | | | | |
| San Diego, US | SD | 26 | 7 | 5.6 | 0 | 0.1 | 8 | 0.769 | 0.397 | | | | | | | | | |

| | | | | | | | | | | | | | | | | | | |
|---|---|---|---|---|---|---|---|---|---|---|---|---|---|---|---|---|---|---|
| *Total sNEP* | | *173* | *16* | | *12* | | *20* | *0.775* | *0.393* | | | | | | | | | |
| *Total NEP* | | *213* | *22* | | *16* | | *21* | *0.814* | *0.419* | | | | | | | | | |
| South Eastern Pacific | SEP | | | | | | | | | | | | | | | | | |
| north SEP | nSEP | | | | | | | | | | | | | | | | | |
| Antofagasta, Chile | Anto | 3 | 3 | - | 0 | - | 3 | 1.000 | 0.346 | | | | | | | | | |
| Coquimbo, Chile | Coqui | 24 | 8 | 6.9 | 2 | 1.3 | 9 | 0.764 | 0.288 | | | | | | | | | This study |
| Guanaqueros, Chile | Guana | 24 | 5 | 3.8 | 1 | 0.6 | 8 | 0.685 | 0.244 | | | | | | | | | |
| *Total nSEP* | | *51* | *12* | | *3* | | *10* | *0.724* | *0.385* | | | | | | | | | |
| south SEP | sSEP | | | | | | | | | | | | | | | | | |
| Talcahuano, Chile | Talca | 9 | 3 | 3.0 | 1 | 1.0 | 4 | 0.556 | 0.25 | | | | | | | | | This study |
| Puerto Montt, Chile | Mont | 13 | 5 | 5.7 | 1 | 1.3 | 4 | 0.808 | 0.231 | | | | | | | | | |
| *Total sSEP* | | *22* | *8* | | *2* | | *5* | *0.771* | *0.246* | | | | | | | | | |
| *Total SEP* | | *73* | *14* | | *6* | | *12* | *0.749* | *0.259* | | | | | | | | | |
| **All dataset** | | | | | | | | | | | | | | | | | | |
| **Mean** | | **21.0** | **3.9** | | | | **4.2** | **0.469** | **0.196** | **25.9** | **8.5** | | | | **14.0** | **0.694** | **0.554** | |
| **(SD)** | | **6.9** | **2.3** | | | | **3.8** | **0.344** | **0.181** | **6.5** | **3.8** | | | | **7.4** | **0.217** | **0.347** | |
| **Total** | | **714** | **45** | | | | **39** | **0.703** | **0.334** | **1140** | **147** | | | | **114** | **0.737** | **0.635** | |

**Table S2. Number of *Ciona robusta* (A) and *C. intestinalis* (B) individuals identified in clusters of the haplotype network based on COX3-ND1 dataset.**

Clusters were identified from the haplotype network built with COX3-ND1 mtDNA sequences and shown in Figure 3 in the main text.

**(A) *Ciona robusta***

| Sampling location | Code | C1 | C2 | Not in cluster | Total |
|---|---|---|---|---|---|
| North Eastern Atlantic | NEA | | | | |
| *English Channel* | EC | | | | |
| Torquay, UK | Tor | 1 | 0 | 0 | 1 |
| Plymouth, UK | Ply | 24 | 0 | 0 | 24 |
| Falmouth, UK | Fal | 24 | 0 | 0 | 24 |
| Saint Vaast, Fr | StV | 24 | 0 | 0 | 24 |
| Saint Malo, Fr | StM | 23 | 0 | 0 | 23 |
| Saint Quay, Fr | StQ | 24 | 0 | 0 | 24 |
| Perros Guirec, Fr | Per | 24 | 0 | 0 | 24 |
| Trébeurden, Fr | Tre | 24 | 0 | 0 | 24 |
| Roscoff-Bloscon, Fr | Blo | 18 | 0 | 0 | 18 |
| Brest-Château, Fr | Cha | 23 | 0 | 0 | 23 |
| Brest-Moulin Blanc, Fr | MBl | 26 | 0 | 0 | 26 |
| Camaret, Fr | Cam | 24 | 0 | 0 | 24 |
| Concarneau, Fr | Con | 24 | 0 | 0 | 24 |
| Crouesty, Fr | Cro | 24 | 0 | 0 | 24 |
| Quiberon, Fr | Qui | 13 | 0 | 0 | 13 |
| *Total EC* | | *320* | *0* | *0* | *320* |
| | | | | | |
| Mediterranean Sea | MedS | | | | |
| Naples, It | Napl | 23 | 0 | 0 | 23 |
| Sete, Fr | Sete | 21 | 0 | 0 | 21 |
| *Total MedS* | | *44* | *0* | *0* | *44* |
| | | | | | |
| North Western Pacific | NWP | | | | |
| Nishinomiya, Japan | Nishi | 29 | 2 | 1 | 32 |
| Tokyo, Japan | Tokyo | 27 | 0 | 5 | 32 |
| *Total NWP* | | *56* | *2* | *6* | *64* |
| | | | | | |
| North Eastern Pacific | NEP | | | | |
| north NEP | nNEP | | | | |
| Tomales Bay, US | TB | 9 | 4 | 0 | 13 |
| San Francisco Estuary, US | SF | 4 | 5 | 0 | 9 |
| Monterey Bay, US | MO | 13 | 5 | 0 | 18 |
| *Total nNEP* | | *26* | *14* | *0* | *40* |
| | | | | | |
| south NEP | sNEP | | | | |
| Santa Barbara, US | SB | 0 | 16 | 0 | 16 |
| Channel Islands, US | CI | 13 | 14 | 0 | 27 |
| Port Hueneme, US | PH | 9 | 12 | 0 | 21 |
| Los Angeles, US | LA | 6 | 25 | 0 | 31 |
| Newport Bay, US | NB | 7 | 14 | 0 | 21 |
| Oceanside Estuary, US | OE | 5 | 18 | 0 | 23 |
| Mission Bay, US | MI | 1 | 7 | 0 | 8 |
| San Diego, US | SD | 11 | 15 | 0 | 26 |
| *Total sNEP* | | *52* | *121* | *0* | *173* |
| | | | | | |
| *Total NEP* | | *78* | *135* | *0* | *213* |
| | | | | | |
| South Eastern Pacific | SEP | | | | |
| north SEP | nSEP | | | | |
| Antofagasta, Chile | Anto | 3 | 0 | 0 | 3 |
| Coquimbo, Chile | Coqui | 19 | 0 | 5 | 24 |
| Guanaqueros, Chile | Guana | 20 | 0 | 4 | 24 |
| *Total nSEP* | | *42* | *0* | *9* | *51* |
| | | | | | |
| south SEP | sSEP | | | | |

| | | | | | |
|---|---|---|---|---|---|
| Talcahuano, Chile | Talca | 9 | 0 | 0 | 9 |
| Puerto Montt, Chile | Mont | 13 | 0 | 0 | 13 |
| *Total sSEP* | | *22* | *0* | *0* | *22* |
| *Total SEP* | | *64* | *0* | *9* | *73* |
| **Total** | | **562** | **137** | **15** | **714** |

## (B) *C. intestinalis*

| **Sampling location** | **Code** | **C1** | **C2** | **C3** | **Not in cluster** | **Total** |
|---|---|---|---|---|---|---|
| North Eastern Atlantic | NEA | | | | | |
| *English Channel* | EC | | | | | |
| Brighton, UK | Bri | 19 | 3 | 1 | 0 | 23 |
| Shoreham, UK | Sho | 16 | 8 | 0 | 0 | 24 |
| Southsea, UK | Shs | 20 | 4 | 0 | 0 | 24 |
| Gosport, UK | Gpt | 16 | 6 | 1 | 0 | 23 |
| Southampton, UK | Sth | 19 | 2 | 0 | 0 | 21 |
| Lymington, UK | Lym | 19 | 3 | 0 | 0 | 22 |
| Poole Quay, UK | Poo | 17 | 7 | 0 | 1 | 24 |
| Torquay, UK | Tor | 16 | 5 | 3 | 0 | 24 |
| Brixham, UK | Brx | 19 | 3 | 2 | 0 | 24 |
| Plymouth, UK | Ply | 13 | 11 | 0 | 0 | 24 |
| Falmouth, UK | Fal | 22 | 1 | 0 | 0 | 23 |
| Saint Vaast, Fr | StV | 15 | 10 | 0 | 0 | 25 |
| Saint Malo, Fr | StM | 12 | 6 | 1 | 0 | 19 |
| Saint Quay, Fr | StQ | 19 | 1 | 4 | 0 | 24 |
| Perros Guirec, Fr | Per | 13 | 13 | 0 | 1 | 26 |
| Trébeurden, Fr | Tre | 13 | 8 | 3 | 0 | 24 |
| Roscoff-Bloscon, Fr | Blo | 18 | 6 | 2 | 0 | 26 |
| Aber Wrac'h, Fr | AbW | 15 | 8 | 2 | 0 | 25 |
| Brest-Château, Fr | Cha | 13 | 11 | 3 | 0 | 27 |
| Brest-Moulin Blanc, Fr | MBl | 15 | 8 | 1 | 0 | 24 |
| Camaret, Fr | Cam | 10 | 8 | 3 | 0 | 21 |
| Concarneau, Fr | Con | 16 | 7 | 1 | 0 | 24 |
| Lorient, Fr | Lor | 11 | 12 | 1 | 0 | 24 |
| Crouesty, Fr | Cro | 13 | 9 | 2 | 0 | 24 |
| Quiberon, Fr | Qui | 19 | 4 | 0 | 0 | 23 |
| *Total EC* | | *398* | *164* | *30* | *2* | *592* |
| *North Sea (Skagerrak)* | *NS* | | | | | |
| Grundsund, Sw | Grun | 21 | 0 | 0 | 0 | 21 |
| Gullmar Fjord, Sw | GullF | 22 | 0 | 0 | 0 | 22 |
| Fiskebäckskil, Sw | Fiske | 24 | 0 | 0 | 0 | 24 |
| *Total NS* | | *67* | *0* | *0* | *0* | *67* |
| Total NEA | | 465 | 164 | 30 | 2 | 661 |
| North Western Atlantic | NWA | | | | | |
| Cardigan River, Ca | CR | 30 | 0 | 0 | 0 | 30 |
| Brudenell River, Ca | BR | 30 | 0 | 0 | 0 | 30 |
| Murray River, Ca | MR | 30 | 0 | 0 | 0 | 30 |
| Sydney, Ca | SD | 41 | 1 | 0 | 0 | 42 |
| Point Tupper, Ca | PO | 21 | 0 | 0 | 0 | 21 |
| Halifax, Ca | HF | 25 | 3 | 0 | 0 | 28 |
| Chester, Ca | CT | 28 | 0 | 0 | 0 | 28 |
| Martin's River, Ca | MA | 45 | 0 | 0 | 0 | 45 |
| Mahone Bay, Ca | MB | 28 | 0 | 0 | 0 | 28 |
| Stone Hurst, Ca | ST | 26 | 0 | 0 | 0 | 26 |
| Lunenburg, Ca | LU | 21 | 0 | 0 | 0 | 21 |
| Shelburne, Ca | SB | 35 | 4 | 0 | 0 | 39 |
| Port La Tour, Ca | PT | 21 | 0 | 0 | 0 | 21 |
| Yarmouth, Ca | YM | 17 | 3 | 0 | 0 | 20 |
| Nahant, US | Nah | 18 | 6 | 0 | 0 | 24 |
| Groton, US | GT | 37 | 11 | 0 | 0 | 48 |
| Total NWA | | 453 | 28 | 0 | 0 | 481 |

| | | | | | | | |
|---|---|---|---|---|---|---|---|
| **Total** | **918** | **192** | **30** | | | **2** | **1140** |

**Table S3. Sampling locations and details of genetic diversity indices of *Ciona robusta* and *C. intestinalis* computed from the concatenated sequences dataset**.

Indices were computed over concatenated mitochondrial DNA sequences (COI and COX3-ND1).

S: syntopic locality (see legend of Supplementary Table S1).

Nind: the number of individuals studied; nf: not found. Indices: Nh: number of haplotypes; Npr: number of private haplotypes; Hd: Haplotype diversity; π: nucleotide diversity.

| | | | ***C. robusta*** | | | | | ***C. intestinalis*** | | | | |
|---|---|---|---|---|---|---|---|---|---|---|---|---|
| **Sampling location** | **Code** | **Status** | **Nind** | **Nh** | **Npr** | **Hd** | **$\pi(10^2)$** | **Nind** | **Nh** | **Npr** | **Hd** | **$\pi(10^2)$** |
| North Eastern Atlantic | NEA | | | | | | | | | | | |
| *English Channel* | EC | | | | | | | | | | | |
| Brighton, UK | Bri | - | nf | | | | | 23 | 15 | 4 | 0.925 | 0.453 |
| Shoreham, UK | Sho | - | nf | | | | | 24 | 18 | 5 | 0.967 | 0.753 |
| Southsea, UK | Shs | - | nf | | | | | 24 | 19 | 10 | 0.978 | 0.528 |
| Gosport, UK | Gpt | - | nf | | | | | 23 | 20 | 9 | 0.976 | 0.718 |
| Southampton, UK | Sth | - | nf | | | | | 21 | 17 | 6 | 0.976 | 0.549 |
| Lymington, UK | Lym | - | nf | | | | | 22 | 18 | 6 | 0.978 | 0.428 |
| Poole Quay, UK | Poo | - | nf | | | | | 24 | 20 | 7 | 0.975 | 0.661 |
| Torquay, UK | Tor | S | 1 | 1 | 0 | - | - | 24 | 11 | 4 | 0.870 | 0.715 |
| Brixham, UK | Brx | - | nf | | | | | 24 | 16 | 4 | 0.946 | 0.607 |
| Plymouth, UK | Ply | S | 24 | 5 | 2 | 0.377 | 0.056 | 24 | 18 | 9 | 0.964 | 0.708 |
| Falmouth, UK | Fal | S | 24 | 5 | 1 | 0.659 | 0.112 | 23 | 15 | 7 | 0.909 | 0.311 |
| Saint Vaast, Fr | StV | S | 24 | 2 | 0 | 0.159 | 0.011 | 25 | 18 | 6 | 0.963 | 0.692 |
| Saint Malo, Fr | StM | S | 23 | 1 | 0 | 0.000 | 0.000 | 19 | 13 | 3 | 0.924 | 0.657 |
| Saint Quay, Fr | StQ | S | 24 | 7 | 4 | 0.667 | 0.078 | 24 | 13 | 7 | 0.931 | 0.592 |
| Perros Guirec, Fr | Per | S | 24 | 3 | 1 | 0.163 | 0.012 | 26 | 21 | 6 | 0.985 | 0.776 |
| Trébeurden, Fr | Tre | S | 24 | 2 | 1 | 0.083 | 0.006 | 24 | 17 | 4 | 0.967 | 0.788 |
| Roscoff-Bloscon, Fr | Blo | S | 18 | 2 | 1 | 0.111 | 0.016 | 26 | 23 | 10 | 0.982 | 0.731 |
| Aber Wrac'h, Fr | AbW | - | nf | | | | | 25 | 19 | 6 | 0.977 | 0.769 |
| Brest-Château, Fr | Cha | S | 23 | 2 | 0 | 0.166 | 0.012 | 27 | 21 | 8 | 0.977 | 0.784 |
| Brest-Moulin Blanc, Fr | MBl | S | 26 | 3 | 1 | 0.151 | 0.011 | 24 | 19 | 9 | 0.975 | 0.729 |
| Camaret, Fr | Cam | S | 24 | 1 | 0 | 0.000 | 0.000 | 21 | 20 | 7 | 0.995 | 0.722 |
| Concarneau, Fr | Con | S | 24 | 2 | 0 | 0.159 | 0.011 | 24 | 16 | 4 | 0.957 | 0.697 |
| Lorient, Fr | Lor | - | nf | | | | | 24 | 19 | 7 | 0.967 | 0.696 |
| Crouesty, Fr | Cro | S | 24 | 2 | 0 | 0.489 | 0.035 | 24 | 19 | 4 | 0.978 | 0.769 |
| Quiberon, Fr | Qui | S | 13 | 4 | 0 | 0.526 | 0.062 | 23 | 18 | 5 | 0.957 | 0.403 |
| *Total EC* | | | *320* | *18* | *13* | *0.285* | *0.033* | *592* | *229* | *157* | *0.979* | *0.692* |
| *North Sea (Skagerrak)* | NS | | | | | | | | | | | |
| Grundsund, Sw | Grun | | | | | | | 21 | 10 | 6 | 0.871 | 0.244 |
| Gullmar Fjord, Sw | GullF | | | | | | | 22 | 10 | 5 | 0.857 | 0.228 |
| Fiskebäckskil, Sw | Fiske | | | | | | | 24 | 10 | 4 | 0.851 | 0.184 |
| *Total NS* | | | | | | | | *67* | *22* | *15* | *0.901* | *0.237* |
| *Total NEA* | | | *320* | *18* | *13* | *0.285* | *0.033* | *659* | *248* | *172* | *0.977* | *0.666* |
| North Western Atlantic | NWA | | | | | | | | | | | |
| Nahant, US | Nah | | | | | | | 24 | 12 | 7 | 0.917 | 0.632 |
| Mediterranean Sea | MedS | | | | | | | | | | | |
| Naples, It | Napl | | 23 | 2 | 0 | 0.166 | 0.012 | | | | | |
| Sete, Fr | Sete | | 21 | 11 | 8 | 0.910 | 0.143 | | | | | |
| *Total MedS* | | | *44* | *11* | *8* | *0.642* | *0.079* | | | | | |
| North Western Pacific | NWP | | | | | | | | | | | |
| Nishinomiya, Japan | Nishi | | 32 | 9 | 2 | 0.845 | 0.454 | | | | | |
| Tokyo, Japan | Tokyo | | 32 | 8 | 1 | 0.774 | 0.34 | | | | | |
| *Total NWP* | | | *64* | *11* | *3* | *0.813* | *0.398* | | | | | |
| South Eastern Pacific | SEP | | | | | | | | | | | |
| *North SEP* | *nSEP* | | | | | | | | | | | |
| Antofagasta, Chile | Anto | | 3 | 3 | 1 | 1.000 | 0.333 | | | | | |
| Coquimbo, Chile | Coqui | | 24 | 13 | 4 | 0.902 | 0.387 | | | | | |
| Guanaqueros, Chile | Guana | | 24 | 12 | 2 | 0.913 | 0.354 | | | | | |
| *Total nSEP* | | | 51 | 17 | 7 | 0.905 | 0.360 | | | | | |
| *South SEP* | *sSEP* | | | | | | | | | | | |
| Talcahuano, Chile | Talca | | 9 | 6 | 2 | 0.833 | 0.345 | | | | | |
| Puerto Montt, Chile | Mont | | 13 | 7 | 1 | 0.846 | 0.397 | | | | | |

| | | | | | | | | | | | |
|---|---|---|---|---|---|---|---|---|---|---|---|
| *Total sSEP* | *sSEP* | *22* | *10* | *3* | *0.879* | *0.404* | | | | | |
| *Total SEP* | | *73* | *21* | *11* | *0.898* | *0.377* | | | | | |
| **All dataset** | | | | | | | | | | | |
| **Mean** | | **21** | **5** | | **0.454** | **0.132** | **24** | **17** | | **0.948** | **0.611** |
| **(SD)** | | **8** | **4** | | **0** | **0** | **2** | **4** | | **0** | **0** |
| **Total** | | **501** | **48** | | **0.598** | **0.225** | **683** | **255** | | **0.977** | **0.665** |

**Table S4. Number of *Ciona intestinalis* individuals identified in clusters C1, C2 and C3 of the haplotype network based on concatenated dataset.**

Clusters were identified from the haplotype network built with concatenated mtDNA sequences (COI and COX3-ND1) and shown in Supplementary Figure S1.

| **Sampling location** | **Code** | **C1** | **C2** | **C3** | **Not in cluster** | **Total** |
|---|---|---|---|---|---|---|
| North Eastern Atlantic | NEA | | | | | |
| *English Channel* | EC | | | | | |
| Brighton, UK | Bri | 19 | 3 | 1 | 0 | 23 |
| Shoreham, UK | Sho | 17 | 7 | 0 | 0 | 24 |
| Southsea, UK | Shs | 20 | 4 | 0 | 0 | 24 |
| Gosport, UK | Gpt | 15 | 6 | 1 | 1 | 23 |
| Southampton, UK | Sth | 17 | 4 | 0 | 0 | 21 |
| Lymington, UK | Lym | 20 | 2 | 0 | 0 | 22 |
| Poole Quay, UK | Poo | 18 | 6 | 0 | 0 | 24 |
| Torquay, UK | Tor | 16 | 5 | 3 | 0 | 24 |
| Brixham, UK | Brx | 20 | 2 | 2 | 0 | 24 |
| Plymouth, UK | Ply | 14 | 9 | 0 | 1 | 24 |
| Falmouth, UK | Fal | 22 | 1 | 0 | 0 | 23 |
| Saint Vaast, Fr | StV | 16 | 9 | 0 | 0 | 25 |
| Saint Malo, Fr | StM | 16 | 2 | 1 | 0 | 19 |
| Saint Quay, Fr | StQ | 18 | 1 | 5 | 0 | 24 |
| Perros Guirec, Fr | Per | 16 | 10 | 0 | 0 | 26 |
| Trébeurden, Fr | Tre | 13 | 8 | 3 | 0 | 24 |
| Roscoff-Bloscon, Fr | Blo | 18 | 4 | 2 | 2 | 26 |
| Aber Wrac'h, Fr | AbW | 18 | 5 | 2 | 0 | 25 |
| Brest-Château, Fr | Cha | 16 | 8 | 3 | 0 | 27 |
| Brest-Moulin Blanc, Fr | MBl | 19 | 4 | 1 | 0 | 24 |
| Camaret, Fr | Cam | 8 | 9 | 4 | 0 | 21 |
| Concarneau, Fr | Con | 16 | 6 | 1 | 1 | 24 |
| Lorient, Fr | Lor | 15 | 8 | 1 | 0 | 24 |
| Crouesty, Fr | Cro | 16 | 6 | 2 | 0 | 24 |
| Quiberon, Fr | Qui | 21 | 2 | 0 | 0 | 23 |
| *Total EC* | | *424* | *131* | *32* | *5* | *592* |
| *North Sea (Skagerrak)* | NS | | | | | |
| Grundsund, Sw | Grun | 21 | 0 | 0 | 0 | 21 |
| Gullmar Fjord, Sw | GullF | 22 | 0 | 0 | 0 | 22 |
| Fiskebäckskil, Sw | Fiske | 24 | 0 | 0 | 0 | 24 |
| *Total NS* | | *67* | *0* | *0* | *0* | *67* |
| Total NEA | | 491 | 131 | 32 | 5 | 659 |
| North Western Atlantic | NWA | | | | | |
| Nahant, US | Nah | 19 | 5 | 0 | 0 | 24 |
| **Total** | | **510** | **136** | **32** | **5** | **683** |

**Table S5. Estimates of pairwise population genetic differentiation for *Ciona robusta* (A) and *C. intestinalis* (B) based on COX3-ND1 sequences.**
The fixation index $\phi_{ST}$ was computed based on COX3-ND1 mitochondrial DNA sequences with the software Arlequin v 3.5. Bold numbers indicate statistical significance (*P*-value <0.05). Population labels are detailed in Supplementary Table S1.

### (A) *C. robusta*

| | Ply | Fal | StV | StM | StQ | Per | Tre | Blo | Cha | MBl | Cam | Con | Cro | Qui | Napl | Set | Coqui | Guana | Talca | Mont | Nishi | Toky | TB | SF | MO | SB | CI | PH | LA | NB | OH |
|---|---|---|---|---|---|---|---|---|---|---|---|---|---|---|---|---|---|---|---|---|---|---|---|---|---|---|---|---|---|---|---|
| Ply | | | | | | | | | | | | | | | | | | | | | | | | | | | | | | | |
| Fal | 0.067 | | | | | | | | | | | | | | | | | | | | | | | | | | | | | | |
| StV | 0.050 | **0.267** | | | | | | | | | | | | | | | | | | | | | | | | | | | | | |
| StM | 0.047 | **0.261** | 0.000 | | | | | | | | | | | | | | | | | | | | | | | | | | | | |
| StQ | **0.067** | **0.215** | 0.087 | 0.084 | | | | | | | | | | | | | | | | | | | | | | | | | | | |
| Per | 0.039 | **0.235** | 0.000 | -0.002 | 0.065 | | | | | | | | | | | | | | | | | | | | | | | | | | |
| Tre | 0.050 | **0.267** | 0.000 | 0.000 | 0.087 | 0.000 | | | | | | | | | | | | | | | | | | | | | | | | | |
| Blo | 0.030 | **0.219** | 0.016 | 0.014 | 0.059 | -0.003 | 0.016 | | | | | | | | | | | | | | | | | | | | | | | | |
| Cha | 0.047 | **0.236** | 0.048 | 0.045 | **0.075** | 0.023 | 0.048 | 0.025 | | | | | | | | | | | | | | | | | | | | | | | |
| MBl | 0.042 | **0.244** | -0.003 | -0.005 | **0.069** | 0.000 | -0.003 | -0.003 | -0.017 | | | | | | | | | | | | | | | | | | | | | | |
| Cam | 0.050 | **0.267** | 0.000 | 0.000 | 0.087 | 0.000 | 0.000 | 0.016 | 0.048 | -0.003 | | | | | | | | | | | | | | | | | | | | | |
| Con | 0.050 | **0.267** | 0.000 | 0.000 | 0.087 | 0.000 | 0.000 | 0.016 | 0.048 | -0.003 | 0.000 | | | | | | | | | | | | | | | | | | | | |
| Cro | 0.050 | **0.267** | 0.000 | 0.000 | 0.087 | 0.000 | 0.000 | 0.016 | 0.048 | -0.003 | 0.000 | 0.000 | | | | | | | | | | | | | | | | | | | |
| Qui | 0.017 | **0.189** | 0.050 | 0.047 | 0.044 | -0.043 | 0.050 | 0.004 | 0.023 | 0.000 | 0.050 | 0.050 | 0.050 | | | | | | | | | | | | | | | | | | |
| Napl | 0.047 | **0.261** | 0.000 | 0.000 | 0.084 | -0.002 | 0.000 | 0.014 | 0.045 | -0.005 | 0.000 | 0.000 | 0.000 | 0.047 | | | | | | | | | | | | | | | | | |
| Sete | 0.034 | **0.221** | 0.007 | 0.004 | 0.059 | 0.000 | 0.007 | -0.038 | -0.025 | -0.021 | 0.007 | 0.007 | 0.007 | -0.003 | 0.004 | | | | | | | | | | | | | | | | |
| Coqui | **0.332** | **0.298** | **0.401** | **0.395** | **0.368** | **0.386** | **0.401** | **0.356** | **0.382** | **0.391** | **0.401** | **0.401** | **0.401** | **0.319** | **0.395** | **0.368** | | | | | | | | | | | | | | | |
| Guana | **0.424** | **0.365** | **0.507** | **0.501** | **0.465** | **0.489** | **0.507** | **0.460** | **0.485** | **0.498** | **0.507** | **0.507** | **0.507** | **0.422** | **0.501** | **0.471** | -0.018 | | | | | | | | | | | | | | |
| Talca | **0.202** | **0.147** | **0.389** | **0.380** | **0.279** | **0.334** | **0.389** | **0.303** | **0.331** | **0.350** | **0.389** | **0.389** | **0.389** | **0.245** | **0.380** | **0.308** | 0.014 | 0.065 | | | | | | | | | | | | | |
| Mont | **0.430** | **0.366** | **0.563** | **0.555** | **0.476** | **0.525** | **0.563** | **0.493** | **0.520** | **0.538** | **0.563** | **0.563** | **0.563** | **0.440** | **0.555** | **0.501** | -0.019 | -0.020 | 0.018 | | | | | | | | | | | | |
| Nishi | **0.439** | **0.395** | **0.496** | **0.491** | **0.470** | **0.484** | **0.496** | **0.458** | **0.481** | **0.494** | **0.496** | **0.496** | **0.496** | **0.426** | **0.491** | **0.469** | **0.044** | 0.016 | **0.110** | 0.013 | | | | | | | | | | | |
| Tokyo | **0.482** | **0.436** | **0.544** | **0.539** | **0.513** | **0.531** | **0.544** | **0.506** | **0.527** | **0.539** | **0.544** | **0.544** | **0.544** | **0.475** | **0.539** | **0.516** | **0.048** | 0.011 | **0.154** | 0.003 | -0.001 | | | | | | | | | | |
| TB | **0.641** | **0.614** | **0.695** | **0.688** | **0.654** | **0.679** | **0.695** | **0.643** | **0.673** | **0.688** | **0.695** | **0.695** | **0.695** | **0.595** | **0.688** | **0.658** | **0.346** | **0.379** | **0.398** | **0.369** | **0.370** | **0.399** | | | | | | | | | |
| SF | **0.670** | **0.638** | **0.732** | **0.725** | **0.684** | **0.713** | **0.732** | **0.675** | **0.707** | **0.723** | **0.732** | **0.732** | **0.732** | **0.621** | **0.725** | **0.691** | **0.328** | **0.366** | **0.387** | **0.350** | **0.347** | **0.371** | -0.033 | | | | | | | | |
| MO | **0.585** | **0.518** | **0.668** | **0.662** | **0.619** | **0.649** | **0.668** | **0.619** | **0.644** | **0.659** | **0.668** | **0.668** | **0.668** | **0.578** | **0.662** | **0.630** | **0.110** | **0.055** | **0.231** | **0.101** | **0.053** | **0.054** | **0.320** | **0.293** | | | | | | | |
| SB | **0.788** | **0.764** | **0.835** | **0.831** | **0.797** | **0.820** | **0.835** | **0.801** | **0.817** | **0.826** | **0.835** | **0.835** | **0.835** | **0.772** | **0.831** | **0.808** | **0.542** | **0.577** | **0.624** | **0.592** | **0.565** | **0.579** | **0.311** | **0.146** | **0.520** | | | | | | |
| CI | **0.468** | **0.445** | **0.507** | **0.501** | **0.486** | **0.498** | **0.507** | **0.467** | **0.493** | **0.504** | **0.507** | **0.507** | **0.507** | **0.431** | **0.501** | **0.481** | **0.186** | **0.199** | **0.244** | **0.188** | **0.220** | **0.209** | **0.132** | 0.035 | **0.160** | **0.186** | | | | | |
| PH | **0.498** | **0.467** | **0.544** | **0.538** | **0.517** | **0.533** | **0.544** | **0.497** | **0.528** | **0.541** | **0.544** | **0.544** | **0.544** | **0.457** | **0.538** | **0.513** | **0.204** | **0.213** | **0.257** | **0.203** | **0.241** | **0.227** | **0.164** | 0.062 | **0.150** | **0.156** | -0.025 | | | | |
| LA | **0.633** | **0.617** | **0.666** | **0.662** | **0.643** | **0.657** | **0.666** | **0.633** | **0.653** | **0.662** | **0.666** | **0.666** | **0.666** | **0.604** | **0.662** | **0.643** | **0.366** | **0.392** | **0.446** | **0.392** | **0.388** | **0.389** | **0.209** | 0.035 | **0.340** | **0.093** | 0.037 | 0.048 | | | |
| NB | **0.595** | **0.569** | **0.642** | **0.637** | **0.610** | **0.629** | **0.642** | **0.598** | **0.625** | **0.636** | **0.642** | **0.642** | **0.642** | **0.560** | **0.637** | **0.612** | **0.272** | **0.298** | **0.356** | **0.295** | **0.298** | **0.297** | **0.174** | 0.024 | **0.252** | **0.161** | -0.015 | 0.003 | -0.021 | | |
| OH | **0.599** | **0.580** | **0.637** | **0.632** | **0.610** | **0.626** | **0.637** | **0.596** | **0.622** | **0.633** | **0.637** | **0.637** | **0.637** | **0.560** | **0.632** | **0.609** | **0.341** | **0.371** | **0.398** | **0.366** | **0.379** | **0.384** | **0.194** | 0.044 | **0.328** | 0.053 | 0.023 | 0.024 | -0.011 | -0.003 | |
| SD | **0.520** | **0.499** | **0.559** | **0.553** | **0.534** | **0.549** | **0.559** | **0.518** | **0.544** | **0.555** | **0.559** | **0.559** | **0.559** | **0.482** | **0.553** | **0.532** | **0.237** | **0.256** | **0.302** | **0.251** | **0.277** | **0.269** | **0.174** | 0.051 | **0.222** | **0.142** | -0.025 | -0.020 | 0.015 | -0.020 | -0.013 |

**(B) *C. intestinalis***

| | Bri | Sho | Shs | Gpt | Sth | Lym | Poo | Tor | Brx | Ply | Fal | StV | StM | StQ | Per | Tre | Blo | AbW | Cha | MBl | Cam | Con | Lor | Cro | Qui | Grun | GullF | Fiske |
|---|---|---|---|---|---|---|---|---|---|---|---|---|---|---|---|---|---|---|---|---|---|---|---|---|---|---|---|---|
| Sho | 0.040 | | | | | | | | | | | | | | | | | | | | | | | | | | | |
| Shs | -0.027 | 0.014 | | | | | | | | | | | | | | | | | | | | | | | | | | |
| Gpt | 0.025 | -0.028 | 0.005 | | | | | | | | | | | | | | | | | | | | | | | | | |
| Sth | -0.020 | 0.004 | -0.024 | -0.013 | | | | | | | | | | | | | | | | | | | | | | | | |
| Lym | -0.024 | 0.078 | -0.013 | 0.061 | -0.002 | | | | | | | | | | | | | | | | | | | | | | | |
| Poo | 0.012 | -0.023 | -0.008 | -0.023 | -0.015 | 0.039 | | | | | | | | | | | | | | | | | | | | | | |
| Tor | 0.051 | 0.026 | 0.037 | 0.015 | 0.039 | **0.088** | 0.025 | | | | | | | | | | | | | | | | | | | | | |
| Brx | 0.011 | 0.052 | 0.015 | 0.033 | 0.021 | 0.036 | 0.037 | -0.001 | | | | | | | | | | | | | | | | | | | | |
| Ply | **0.133** | -0.005 | **0.090** | 0.008 | **0.080** | **0.178** | 0.021 | 0.049 | **0.118** | | | | | | | | | | | | | | | | | | | |
| Fal | 0.049 | **0.162** | 0.056 | **0.140** | 0.074 | 0.031 | **0.126** | **0.163** | **0.091** | **0.270** | | | | | | | | | | | | | | | | | | |
| StV | 0.057 | -0.030 | 0.030 | -0.022 | 0.019 | **0.098** | -0.009 | 0.031 | **0.058** | -0.010 | **0.175** | | | | | | | | | | | | | | | | | |
| StM | 0.007 | -0.031 | -0.012 | -0.038 | -0.018 | 0.049 | -0.034 | -0.011 | -0.002 | 0.015 | **0.140** | -0.021 | | | | | | | | | | | | | | | | |
| StQ | 0.047 | **0.097** | 0.046 | **0.067** | 0.056 | **0.067** | **0.076** | -0.003 | -0.013 | **0.155** | **0.133** | **0.106** | 0.037 | | | | | | | | | | | | | | | |
| Per | **0.129** | -0.007 | **0.088** | 0.004 | 0.077 | **0.174** | 0.021 | 0.062 | **0.117** | -0.024 | **0.260** | -0.018 | 0.011 | **0.164** | | | | | | | | | | | | | | |
| Tre | **0.092** | -0.005 | 0.065 | -0.014 | 0.049 | **0.141** | 0.013 | 0.023 | **0.057** | 0.002 | **0.220** | -0.009 | -0.019 | **0.095** | -0.008 | | | | | | | | | | | | | |
| Blo | 0.002 | -0.017 | -0.008 | -0.024 | -0.014 | 0.036 | -0.025 | 0.009 | 0.012 | 0.029 | **0.094** | -0.007 | -0.040 | 0.046 | 0.026 | -0.001 | | | | | | | | | | | | |
| AbW | 0.051 | -0.027 | 0.028 | -0.026 | 0.016 | **0.092** | -0.016 | 0.019 | 0.049 | -0.004 | **0.160** | -0.023 | -0.032 | **0.086** | -0.010 | -0.021 | -0.019 | | | | | | | | | | | |
| Cha | **0.128** | 0.002 | **0.092** | 0.004 | **0.082** | **0.180** | 0.023 | 0.053 | **0.105** | -0.014 | **0.267** | 0.001 | 0.005 | **0.144** | -0.015 | -0.023 | 0.019 | -0.014 | | | | | | | | | | |
| MBl | 0.003 | -0.025 | -0.012 | -0.035 | -0.024 | 0.036 | -0.031 | 0.012 | 0.013 | 0.022 | **0.117** | -0.018 | -0.042 | 0.050 | 0.020 | -0.003 | -0.032 | -0.023 | 0.017 | | | | | | | | | |
| Cam | **0.176** | 0.034 | **0.136** | 0.036 | **0.128** | **0.227** | 0.068 | **0.075** | **0.137** | -0.002 | **0.309** | 0.016 | 0.042 | **0.170** | -0.008 | -0.010 | 0.053 | 0.013 | -0.020 | 0.052 | | | | | | | | |
| Con | **0.150** | 0.001 | **0.106** | 0.011 | 0.094 | **0.201** | 0.030 | 0.070 | **0.139** | -0.032 | **0.297** | -0.005 | 0.025 | **0.177** | -0.026 | -0.003 | 0.034 | -0.003 | -0.022 | 0.027 | -0.011 | | | | | | | |
| Lor | **0.189** | 0.017 | **0.140** | 0.031 | **0.125** | **0.241** | 0.052 | **0.091** | **0.161** | -0.025 | **0.334** | 0.008 | 0.043 | **0.206** | -0.020 | 0.003 | 0.056 | 0.008 | -0.021 | 0.050 | -0.016 | -0.029 | | | | | | |
| Cro | **0.096** | -0.019 | 0.065 | -0.016 | 0.052 | **0.148** | 0.004 | 0.034 | **0.078** | -0.021 | **0.234** | -0.024 | -0.016 | **0.123** | -0.027 | -0.031 | -0.002 | -0.026 | -0.029 | -0.006 | -0.017 | -0.025 | -0.018 | | | | | |
| Qui | -0.026 | 0.048 | -0.020 | 0.034 | -0.018 | -0.020 | 0.020 | 0.068 | 0.020 | **0.149** | 0.045 | **0.067** | 0.014 | **0.058** | **0.141** | **0.105** | 0.013 | 0.059 | **0.146** | 0.011 | **0.195** | **0.169** | **0.202** | **0.112** | | | | |
| Grun | **0.089** | **0.210** | **0.096** | **0.186** | **0.114** | **0.071** | **0.175** | **0.204** | **0.128** | **0.315** | **0.125** | **0.218** | **0.197** | **0.165** | **0.301** | **0.264** | **0.148** | **0.217** | **0.313** | **0.164** | **0.348** | **0.344** | **0.381** | **0.281** | **0.073** | | | |
| GullF | **0.060** | **0.195** | **0.068** | **0.169** | **0.094** | 0.026 | **0.156** | **0.189** | **0.107** | **0.305** | **0.077** | **0.204** | **0.177** | **0.136** | **0.297** | **0.253** | **0.131** | **0.203** | **0.302** | **0.140** | **0.341** | **0.333** | **0.372** | **0.270** | 0.051 | **0.049** | | |
| Fiske | **0.063** | **0.201** | **0.069** | **0.174** | **0.095** | 0.026 | **0.161** | **0.197** | **0.113** | **0.313** | **0.082** | **0.213** | **0.186** | **0.139** | **0.305** | **0.262** | **0.136** | **0.210** | **0.310** | **0.146** | **0.351** | **0.339** | **0.382** | **0.279** | 0.050 | **0.047** | -0.018 | |
| CR | 0.039 | **0.195** | **0.062** | **0.166** | **0.086** | 0.024 | **0.149** | **0.186** | **0.100** | **0.315** | **0.081** | **0.208** | **0.172** | **0.138** | **0.301** | **0.254** | **0.121** | **0.200** | **0.305** | **0.140** | **0.355** | **0.344** | **0.384** | **0.272** | 0.036 | **0.094** | **0.054** | **0.060** |
| BR | **0.086** | **0.254** | **0.100** | **0.224** | **0.138** | **0.048** | **0.209** | **0.246** | **0.145** | **0.377** | **0.114** | **0.262** | **0.259** | **0.205** | **0.364** | **0.318** | **0.172** | **0.260** | **0.369** | **0.202** | **0.418** | **0.411** | **0.453** | **0.339** | **0.080** | **0.159** | **0.052** | **0.058** |
| MR | **0.079** | **0.248** | **0.095** | **0.218** | **0.132** | **0.043** | **0.202** | **0.240** | **0.139** | **0.372** | **0.110** | **0.257** | **0.251** | **0.197** | **0.359** | **0.312** | **0.166** | **0.254** | **0.363** | **0.195** | **0.413** | **0.406** | **0.447** | **0.333** | **0.074** | **0.159** | **0.052** | **0.058** |
| SD | 0.038 | **0.202** | **0.063** | **0.173** | **0.087** | 0.024 | **0.153** | **0.197** | **0.108** | **0.332** | **0.091** | **0.219** | **0.176** | **0.147** | **0.319** | **0.268** | **0.126** | **0.209** | **0.319** | **0.144** | **0.379** | **0.361** | **0.401** | **0.286** | 0.035 | **0.135** | **0.057** | **0.063** |
| PO | 0.045 | **0.197** | 0.062 | **0.168** | 0.091 | 0.018 | **0.155** | **0.189** | **0.100** | **0.315** | **0.082** | **0.207** | **0.189** | **0.145** | **0.304** | **0.256** | **0.123** | **0.203** | **0.308** | **0.145** | **0.350** | **0.347** | **0.388** | **0.276** | 0.042 | **0.129** | 0.038 | **0.043** |
| HF | -0.020 | 0.069 | -0.009 | 0.050 | -0.005 | -0.006 | 0.032 | **0.085** | 0.037 | **0.176** | 0.068 | **0.089** | 0.031 | **0.079** | **0.168** | **0.128** | 0.024 | **0.079** | **0.170** | 0.027 | **0.227** | **0.200** | **0.235** | **0.135** | -0.021 | **0.128** | **0.085** | **0.091** |
| CT | **0.049** | **0.204** | **0.071** | **0.174** | **0.097** | **0.031** | **0.160** | **0.194** | **0.107** | **0.323** | **0.091** | **0.216** | **0.187** | **0.145** | **0.312** | **0.263** | **0.129** | **0.209** | **0.313** | **0.150** | **0.361** | **0.353** | **0.393** | **0.282** | 0.050 | **0.133** | **0.060** | **0.066** |
| MA | **0.068** | **0.217** | **0.091** | **0.191** | **0.115** | **0.055** | **0.170** | **0.208** | **0.127** | **0.334** | **0.102** | **0.233** | **0.188** | **0.149** | **0.324** | **0.275** | **0.147** | **0.223** | **0.325** | **0.158** | **0.377** | **0.358** | **0.397** | **0.291** | **0.069** | **0.118** | **0.050** | **0.058** |
| MB | **0.111** | **0.214** | **0.116** | **0.194** | **0.141** | **0.100** | **0.178** | **0.211** | **0.147** | **0.312** | **0.159** | **0.224** | **0.198** | **0.166** | **0.306** | **0.262** | **0.157** | **0.219** | **0.310** | **0.162** | **0.345** | **0.334** | **0.374** | **0.277** | **0.114** | **0.169** | **0.099** | **0.109** |
| ST | **0.117** | **0.212** | **0.119** | **0.193** | **0.144** | **0.106** | **0.179** | **0.210** | **0.149** | **0.307** | **0.163** | **0.222** | **0.197** | **0.167** | **0.301** | **0.259** | **0.158** | **0.218** | **0.306** | **0.162** | **0.338** | **0.328** | **0.367** | **0.274** | **0.119** | **0.163** | **0.104** | **0.114** |
| LU | 0.031 | **0.176** | 0.049 | **0.148** | 0.075 | 0.016 | **0.134** | **0.170** | **0.088** | **0.292** | **0.087** | **0.187** | **0.161** | **0.128** | **0.281** | **0.233** | **0.105** | **0.181** | **0.284** | **0.124** | **0.328** | **0.323** | **0.363** | **0.252** | 0.032 | **0.142** | **0.061** | **0.066** |
| SB | -0.009 | **0.093** | 0.003 | **0.075** | 0.009 | -0.008 | 0.053 | **0.106** | 0.048 | **0.206** | 0.048 | **0.115** | 0.054 | **0.087** | **0.195** | **0.157** | 0.046 | **0.106** | **0.201** | 0.047 | **0.261** | **0.229** | **0.264** | **0.165** | -0.018 | **0.072** | **0.049** | **0.054** |
| PT | 0.046 | **0.200** | 0.063 | 0.171 | 0.093 | 0.019 | **0.158** | **0.192** | **0.102** | **0.318** | **0.086** | **0.209** | **0.194** | **0.148** | **0.307** | **0.259** | **0.125** | **0.205** | **0.311** | **0.148** | **0.353** | **0.350** | **0.392** | **0.279** | 0.044 | **0.138** | 0.040 | **0.046** |
| YM | 0.021 | 0.030 | 0.007 | 0.028 | 0.010 | 0.042 | 0.011 | 0.065 | 0.051 | **0.097** | **0.117** | 0.043 | 0.012 | **0.088** | **0.092** | **0.074** | 0.015 | 0.040 | **0.103** | 0.009 | **0.143** | **0.113** | **0.140** | **0.073** | 0.021 | **0.153** | **0.129** | **0.137** |
| Nah | 0.022 | 0.025 | 0.002 | 0.023 | 0.010 | 0.058 | 0.029 | 0.028 | 0.043 | **0.018** | **0.142** | 0.011 | 0.034 | **0.088** | 0.016 | 0.010 | 0.002 | 0.018 | 0.023 | 0.028 | **0.067** | **0.028** | **0.047** | 0.002 | 0.026 | **0.184** | **0.174** | **0.183** |
| GT | 0.017 | 0.018 | 0.011 | 0.018 | 0.013 | **0.045** | 0.006 | **0.054** | **0.056** | **0.078** | **0.109** | 0.028 | 0.005 | **0.090** | **0.074** | **0.062** | 0.008 | 0.030 | **0.089** | 0.002 | **0.129** | **0.086** | **0.121** | 0.052 | 0.027 | **0.144** | **0.118** | **0.124** |

## Table S5b *C. intestinalis* (contined)

| | CR | BR | MR | SD | PO | HF | CT | MA | MB | ST | LU | SB | PT | YM | Nah |
|---|---|---|---|---|---|---|---|---|---|---|---|---|---|---|---|
| Sho | | | | | | | | | | | | | | | |
| Shs | | | | | | | | | | | | | | | |
| Gpt | | | | | | | | | | | | | | | |
| Sth | | | | | | | | | | | | | | | |
| Lym | | | | | | | | | | | | | | | |
| Poo | | | | | | | | | | | | | | | |
| Tor | | | | | | | | | | | | | | | |
| Brx | | | | | | | | | | | | | | | |
| Ply | | | | | | | | | | | | | | | |
| Fal | | | | | | | | | | | | | | | |
| StV | | | | | | | | | | | | | | | |
| StM | | | | | | | | | | | | | | | |
| StQ | | | | | | | | | | | | | | | |
| Per | | | | | | | | | | | | | | | |
| Tre | | | | | | | | | | | | | | | |
| Blo | | | | | | | | | | | | | | | |
| AbW | | | | | | | | | | | | | | | |
| Cha | | | | | | | | | | | | | | | |
| MBl | | | | | | | | | | | | | | | |
| Cam | | | | | | | | | | | | | | | |
| Con | | | | | | | | | | | | | | | |
| Lor | | | | | | | | | | | | | | | |
| Cro | | | | | | | | | | | | | | | |
| Qui | | | | | | | | | | | | | | | |
| Grun | | | | | | | | | | | | | | | |
| GullF | | | | | | | | | | | | | | | |
| Fiske | | | | | | | | | | | | | | | |
| CR | | | | | | | | | | | | | | | |
| BR | **0.056** | | | | | | | | | | | | | | |
| MR | 0.036 | 0.023 | | | | | | | | | | | | | |
| SD | -0.004 | **0.072** | **0.050** | | | | | | | | | | | | |
| PO | -0.006 | 0.006 | 0.006 | 0.002 | | | | | | | | | | | |
| HF | 0.032 | **0.115** | **0.100** | 0.018 | 0.054 | | | | | | | | | | |
| CT | -0.011 | **0.071** | **0.046** | -0.006 | -0.005 | 0.043 | | | | | | | | | |
| MA | 0.036 | **0.087** | **0.078** | 0.026 | 0.042 | **0.068** | 0.026 | | | | | | | | |
| MB | **0.136** | **0.199** | **0.193** | **0.129** | **0.151** | **0.139** | **0.128** | 0.009 | | | | | | | |
| ST | **0.147** | **0.207** | **0.202** | **0.147** | **0.161** | **0.150** | **0.142** | 0.020 | -0.033 | | | | | | |
| LU | -0.021 | **0.102** | 0.065 | -0.024 | -0.009 | 0.021 | -0.022 | 0.032 | **0.143** | **0.156** | | | | | |
| SB | 0.011 | **0.058** | **0.050** | 0.018 | 0.025 | -0.015 | 0.032 | **0.057** | **0.114** | **0.122** | 0.015 | | | | |
| PT | -0.007 | 0.035 | 0.003 | 0.001 | -0.039 | 0.055 | -0.007 | 0.044 | **0.156** | **0.166** | -0.010 | 0.025 | | | |
| YM | **0.121** | **0.199** | **0.192** | **0.130** | **0.142** | 0.031 | **0.138** | **0.116** | **0.115** | **0.115** | **0.114** | 0.043 | **0.145** | | |
| Nah | **0.162** | **0.232** | **0.226** | **0.173** | **0.177** | 0.041 | **0.180** | **0.189** | **0.197** | **0.195** | **0.155** | 0.056 | **0.180** | 0.009 | |
| GT | **0.114** | **0.152** | **0.147** | **0.110** | **0.117** | 0.037 | **0.118** | **0.099** | **0.090** | **0.093** | **0.103** | **0.051** | **0.118** | 0.009 | 0.008 |

**Table S6. Estimates of pairwise population genetic differentiation for *Ciona robusta* (A) and *C. intestinalis* (B) based on concatenated mitochondrial DNA sequences.**

The fixation index $\phi_{ST}$ was computed based on concatenated mitochondrial DNA sequences (COI and COX3-ND1) with the software Arlequin v 3.5. Bold numbers indicate statistical significance (*P*-value <0.05). Population labels are detailed in Table S1.

**(A) *C. robusta***

| | Blo | Cha | MBl | Cam | Con | Cro | Fal | Per | Ply | StQ | StM | Tre | StV | Guana | Coqui | Nishi | Tokyo | Napl |
|---|---|---|---|---|---|---|---|---|---|---|---|---|---|---|---|---|---|---|
| Blo | | | | | | | | | | | | | | | | | | |
| Cha | 0.022 | | | | | | | | | | | | | | | | | |
| MBl | 0.003 | -0.017 | | | | | | | | | | | | | | | | |
| Cam | 0.016 | 0.048 | -0.003 | | | | | | | | | | | | | | | |
| Con | -0.029 | 0.045 | 0.023 | 0.043 | | | | | | | | | | | | | | |
| Cro | **0.188** | **0.288** | **0.294** | **0.348** | **0.181** | | | | | | | | | | | | | |
| Fal | **0.224** | **0.253** | **0.264** | **0.274** | **0.258** | **0.293** | | | | | | | | | | | | |
| Per | 0.002 | 0.023 | 0.000 | 0.000 | 0.022 | **0.285** | **0.255** | | | | | | | | | | | |
| Ply | 0.038 | 0.056 | 0.055 | 0.061 | 0.058 | **0.197** | 0.078 | 0.051 | | | | | | | | | | |
| StQ | 0.040 | **0.069** | **0.071** | **0.079** | **0.058** | **0.147** | **0.187** | **0.066** | **0.064** | | | | | | | | | |
| StM | 0.014 | 0.045 | -0.005 | 0.000 | 0.041 | **0.342** | **0.269** | -0.002 | 0.058 | **0.076** | | | | | | | | |
| Tre | 0.007 | 0.032 | 0.000 | 0.000 | 0.029 | **0.313** | **0.264** | 0.000 | 0.055 | **0.072** | -0.002 | | | | | | | |
| StV | -0.029 | 0.045 | 0.000 | 0.043 | -0.043 | **0.181** | **0.258** | 0.022 | 0.058 | **0.058** | 0.041 | 0.029 | | | | | | |
| Guana | **0.460** | **0.494** | **0.511** | **0.507** | **0.498** | **0.487** | **0.405** | **0.499** | **0.452** | **0.437** | **0.501** | **0.503** | **0.498** | | | | | |
| Coqui | **0.364** | **0.399** | **0.416** | **0.412** | **0.401** | **0.391** | **0.325** | **0.404** | **0.361** | **0.349** | **0.406** | **0.408** | **0.401** | -0.014 | | | | |
| Nishi | **0.508** | **0.535** | **0.549** | **0.544** | **0.540** | **0.538** | **0.451** | **0.540** | **0.499** | **0.522** | **0.539** | **0.542** | **0.540** | **0.056** | **0.088** | | | |
| Tokyo | **0.575** | **0.601** | **0.615** | **0.611** | **0.606** | **0.602** | **0.509** | **0.560** | **0.562** | **0.584** | **0.606** | **0.608** | **0.606** | **0.071** | **0.122** | 0.006 | | |
| Napl | 0.000 | 0.036 | 0.021 | 0.033 | -0.034 | 0.156 | **0.244** | 0.019 | 0.052 | **0.051** | 0.030 | 0.024 | -0.034 | **0.488** | **0.392** | **0.535** | **0.601** | |
| Sete | **0.103** | **0.151** | **0.163** | **0.171** | **0.120** | 0.056 | **0.214** | **0.157** | **0.137** | **0.104** | **0.166** | **0.164** | **0.120** | **0.425** | **0.338** | **0.497** | **0.555** | **0.111** |

## (B) *C. intestinalis*

| | AbW | Blo | Cha | Bri | MBl | Brx | Cam | Con | Cro | Fal | Gpt | Lor | Lym | Per | Ply | Poo | Qui | Sho | Shs | StQ | Sth | StM | Tre | Tor | StV | Fiske | Grun | GullF |
|---|---|---|---|---|---|---|---|---|---|---|---|---|---|---|---|---|---|---|---|---|---|---|---|---|---|---|---|---|
| AbW | | | | | | | | | | | | | | | | | | | | | | | | | | | | |
| Blo | -0.012 | | | | | | | | | | | | | | | | | | | | | | | | | | | |
| Cha | -0.025 | -0.015 | | | | | | | | | | | | | | | | | | | | | | | | | | |
| Bri | 0.052 | 0.002 | 0.048 | | | | | | | | | | | | | | | | | | | | | | | | | |
| MBl | 0.028 | -0.010 | -0.021 | 0.050 | | | | | | | | | | | | | | | | | | | | | | | | |
| Brx | 0.045 | 0.006 | 0.029 | 0.013 | 0.045 | | | | | | | | | | | | | | | | | | | | | | | |
| Cam | 0.016 | **0.069** | 0.014 | **0.175** | 0.027 | **0.139** | | | | | | | | | | | | | | | | | | | | | | |
| Con | -0.009 | 0.036 | 0.000 | **0.130** | -0.014 | **0.117** | -0.005 | | | | | | | | | | | | | | | | | | | | | |
| Cro | -0.021 | 0.012 | -0.025 | **0.092** | -0.019 | **0.073** | -0.012 | -0.028 | | | | | | | | | | | | | | | | | | | | |
| Fal | **0.162** | **0.099** | **0.190** | **0.089** | **0.182** | **0.106** | **0.312** | **0.276** | **0.228** | | | | | | | | | | | | | | | | | | | |
| Gpt | -0.022 | -0.020 | -0.020 | 0.021 | -0.026 | 0.024 | 0.050 | 0.009 | -0.008 | 0.145 | | | | | | | | | | | | | | | | | | |
| Lor | 0.005 | **0.064** | 0.012 | **0.172** | 0.006 | **0.145** | -0.015 | -0.029 | -0.020 | 0.316 | 0.034 | | | | | | | | | | | | | | | | | |
| Lym | **0.094** | 0.035 | **0.097** | 0.002 | **0.091** | 0.037 | **0.229** | **0.182** | **0.142** | **0.078** | **0.049** | **0.225** | | | | | | | | | | | | | | | | |
| Nah | -0.008 | -0.009 | 0.000 | 0.035 | -0.008 | **0.034** | **0.080** | 0.027 | 0.011 | 0.132 | -0.012 | 0.050 | 0.068 | | | | | | | | | | | | | | | |
| Per | -0.009 | 0.035 | 0.004 | **0.115** | -0.011 | **0.103** | 0.002 | -0.027 | -0.022 | **0.250** | 0.009 | -0.020 | **0.162** | | | | | | | | | | | | | | | |
| Ply | -0.009 | 0.029 | 0.006 | **0.112** | -0.013 | 0.092 | 0.013 | -0.027 | -0.016 | **0.255** | 0.005 | -0.015 | **0.162** | -0.022 | | | | | | | | | | | | | | |
| Poo | -0.011 | -0.018 | -0.011 | 0.012 | -0.017 | 0.027 | **0.075** | 0.024 | 0.009 | **0.135** | -0.021 | 0.049 | 0.042 | 0.017 | 0.014 | | | | | | | | | | | | | |
| Qui | 0.067 | 0.014 | 0.072 | -0.006 | 0.066 | 0.015 | 0.205 | **0.154** | **0.113** | **0.078** | 0.035 | **0.194** | -0.001 | **0.133** | **0.133** | 0.022 | | | | | | | | | | | | |
| Sho | -0.021 | -0.010 | -0.015 | 0.044 | -0.022 | 0.045 | 0.040 | -0.002 | -0.014 | **0.165** | -0.024 | 0.018 | **0.083** | -0.004 | -0.005 | -0.013 | 0.053 | | | | | | | | | | | |
| Shs | 0.036 | -0.006 | 0.031 | -0.016 | 0.030 | 0.012 | **0.153** | **0.099** | 0.069 | **0.082** | 0.005 | **0.140** | -0.003 | **0.088** | **0.085** | -0.003 | 0.000 | 0.018 | | | | | | | | | | |
| StQ | **0.077** | 0.037 | 0.058 | 0.058 | **0.083** | -0.005 | **0.156** | **0.154** | **0.108** | **0.155** | **0.063** | **0.183** | **0.086** | **0.148** | **0.132** | **0.074** | **0.076** | **0.090** | **0.062** | | | | | | | | | |
| Sth | 0.027 | -0.009 | 0.030 | -0.006 | 0.020 | 0.020 | **0.144** | **0.092** | 0.063 | **0.099** | -0.007 | **0.129** | -0.005 | **0.079** | **0.077** | -0.012 | -0.006 | 0.019 | -0.019 | **0.068** | | | | | | | | |
| StM | -0.027 | -0.030 | -0.028 | 0.014 | -0.027 | 0.009 | 0.051 | 0.012 | -0.010 | **0.147** | -0.031 | 0.035 | **0.056** | 0.009 | 0.004 | -0.030 | 0.027 | -0.028 | -0.002 | 0.048 | -0.007 | | | | | | | |
| Tre | -0.019 | 0.007 | -0.024 | **0.086** | -0.018 | 0.055 | -0.004 | -0.011 | -0.026 | **0.218** | -0.007 | -0.003 | **0.135** | -0.011 | -0.003 | 0.011 | **0.103** | -0.003 | **0.067** | **0.081** | 0.055 | -0.012 | | | | | | |
| Tor | 0.024 | 0.016 | 0.012 | 0.060 | 0.028 | 0.003 | **0.077** | 0.061 | 0.032 | **0.172** | 0.018 | **0.082** | **0.097** | 0.061 | 0.039 | 0.029 | **0.078** | 0.024 | 0.047 | 0.005 | 0.050 | 0.002 | 0.024 | | | | | |
| StV | -0.020 | 0.002 | -0.007 | 0.053 | -0.023 | 0.055 | 0.031 | -0.008 | -0.018 | **0.192** | -0.018 | 0.011 | **0.101** | -0.015 | -0.013 | -0.010 | **0.072** | -0.021 | 0.033 | **0.107** | 0.030 | -0.017 | -0.008 | 0.038 | | | | |
| Fiske | **0.220** | **0.139** | **0.251** | **0.111** | **0.226** | **0.135** | **0.371** | **0.336** | **0.284** | **0.060** | **0.187** | **0.381** | **0.085** | **0.305** | **0.313** | **0.174** | **0.095** | **0.213** | **0.103** | **0.181** | **0.125** | **0.199** | **0.270** | **0.217** | **0.241** | | | |
| Grun | **0.241** | **0.178** | **0.273** | **0.178** | **0.255** | **0.181** | **0.372** | **0.349** | **0.301** | **0.098** | **0.217** | **0.387** | **0.148** | **0.315** | **0.328** | **0.213** | **0.168** | **0.240** | **0.167** | **0.223** | **0.174** | **0.232** | **0.287** | **0.244** | **0.266** | **0.091** | | |
| GullF | **0.200** | **0.114** | **0.218** | **0.072** | **0.198** | **0.103** | **0.344** | **0.309** | **0.257** | **0.117** | **0.153** | **0.353** | 0.026 | **0.277** | **0.285** | **0.140** | **0.054** | **0.184** | **0.066** | **0.150** | **0.072** | **0.163** | **0.240** | **0.186** | **0.212** | **0.075** | **0.188** | |
| Nah | -0.008 | -0.009 | 0.000 | 0.035 | -0.008 | **0.034** | **0.080** | 0.027 | 0.011 | 0.132 | -0.012 | 0.050 | 0.068 | 0.020 | 0.019 | -0.013 | 0.026 | -0.015 | 0.009 | **0.091** | 0.012 | -0.020 | 0.014 | 0.037 | -0.004 | **0.182** | **0.204** | **0.169** |

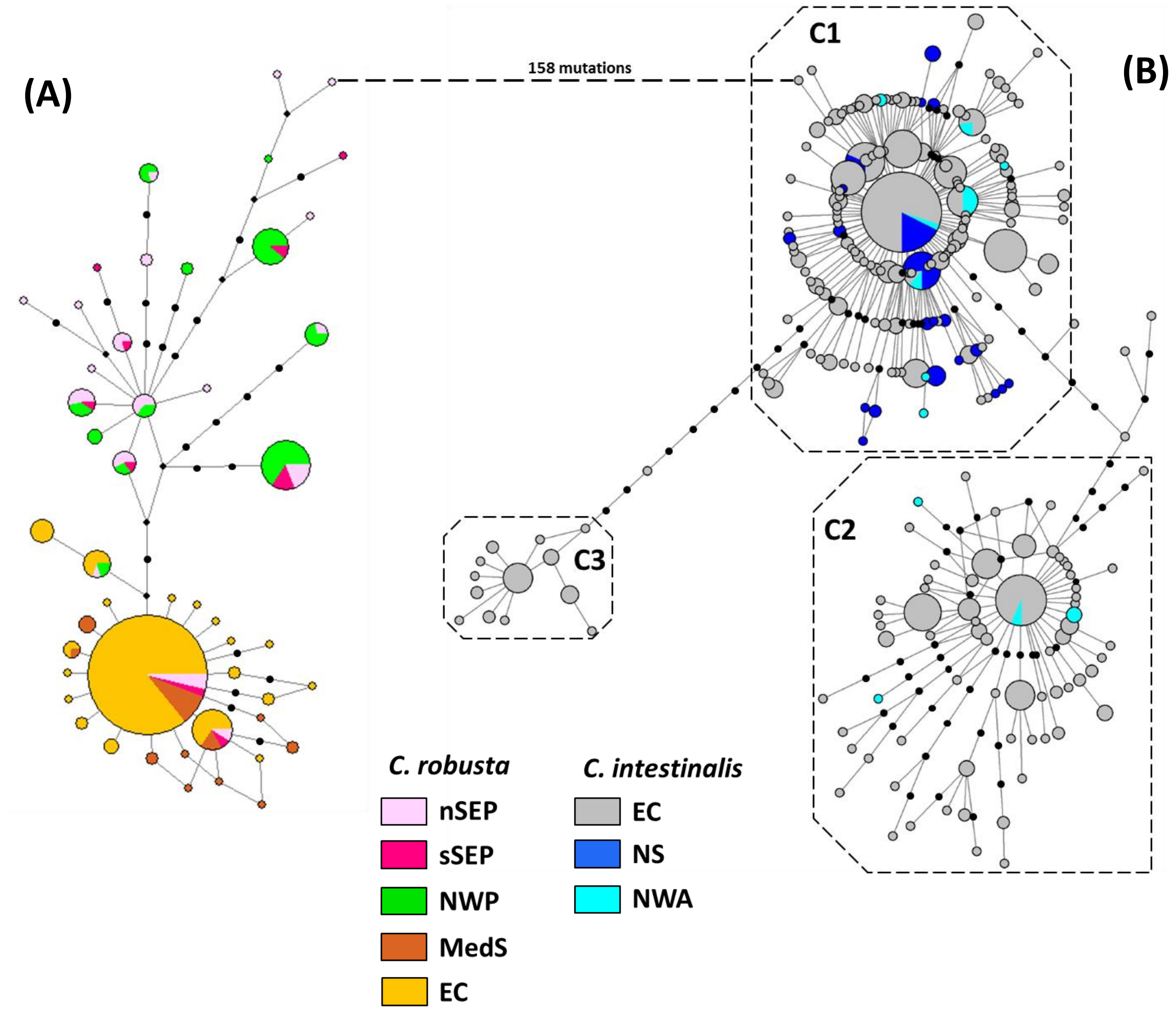


**Supplementary Figure S1. Median-joining haplotype networks of *Ciona robusta* (a) and *C. intestinalis* (b) based on concatenated mtDNA sequences.**

Haplotype circles are proportional to haplotype frequency in the whole dataset. Branch lengths are proportional to number of mutational steps between two haplotypes. Missing haplotypes are indicated by small black circles. Colors represent the location of individuals possessing the haplotypes. A dotted line gives the number of mutations between the most similar haplotypes in the two species.

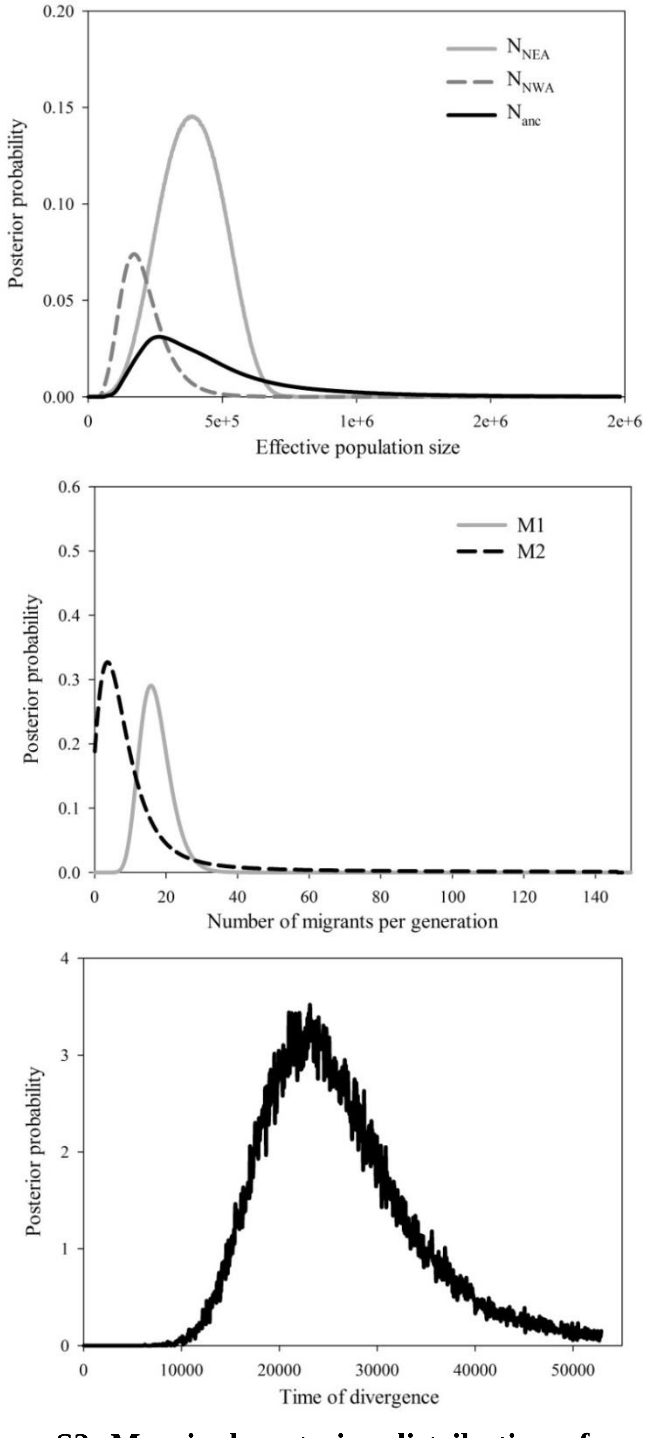


**Supplementary Figure S2. Marginal posterior distribution of parameters in the IMM computed with IMa2 for examining the history of isolation between populations of *C. intestinalis*.**

Distribution curves are shown for 1) the effective size of the study populations, namely NE Atlantic and NW Atlantic ($N_{NEA}$ and $N_{NWA}$, respectively) and their ancestral population ($N_{anc}$), 2) the number of effective migrants per generation (with $M_1$ and $M_2$ being the number of migrants from $N_{NEA}$ to $N_{NWA}$ and from $N_{NWA}$ to $N_{NEA}$, respectively) and 3) the time of divergence between the two sides of the Atlantic. The median value and the 95% highest posterior density of each parameter are given in Figure 5 in the main text.
